# Supplementary material for: Development and Assessment of 1,5–Diarylpyrazole/Oxime Hybrids Targeting EGFR and JNK–2 as Antiproliferative Agents: A Comprehensive Study through Synthesis, Molecular Docking, and Evaluation
Source: Molecules. 2023 Sep 8;28(18):6521. doi: 10.3390/molecules28186521 (PMC10537604; doi:10.3390/molecules28186521)
Supplement: Supplementary file 1 [file molecules-28-06521-s001.zip › molecules-2602124-supplementary.pdf]

Supplementary information

Development and Assessment of 1,5-Diarylpyrazole/Oxime Hybrids Targeting EGFR and JNK-2 as Antiproliferative Agents: A Comprehensive Study Through Synthesis, Molecular Docking, and Evaluation

Kamal S. Abdelrahman[a,*], Heba A. Hassan[b], Salah A. Abdel-Aziz[a,c], Adel A. Marzouk[a,d], Raef Shams[e] , Keima Osawa[f], Mohamed Abdel-Aziz[b], and Hiroyuki Konno[f,*]

[a] Department of Pharmaceutical Chemistry, Faculty of Pharmacy, Al-Azhar University, Assiut Branch, Assiut 71524, Egypt

[b] Department of Medicinal Chemistry Faculty of Pharmacy, Minia University, Minia 61519, Egypt

[c] Department of Pharmaceutical Chemistry, Faculty of Pharmacy, Deraya University, Minia, Egypt

[d] National Center for Natural Products Research, School of Pharmacy, University of Missippi, Oxford, MS 38677, USA

[e] Emergent Bioengineering Materials Research Team, RIKEN Centre for Emergent Matter Science, RIKEN, Wako, Saitama 351-0198, Japan

[f] Graduate School of Science and Engineering, Yamagata University, Yonezawa, Yamagata 992-8510, Japan

* Correspondance: kamal_kamal20082002@yahoo.com (K.S.A); konno@yz.yamagata-u.ac.jp (H.K.), Tel. & FAX: +81-238-26-3131.

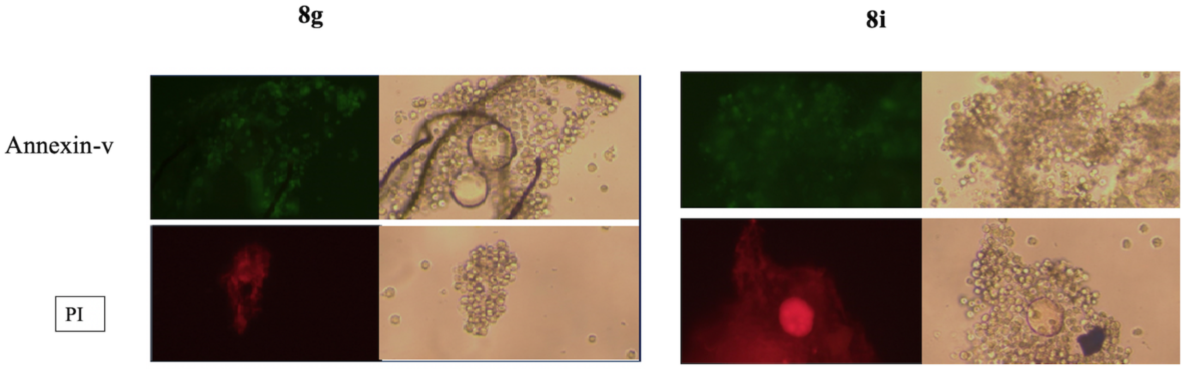

Figure S1. Microscopic examination of apoptosis and necrosis of compounds **8g** and **8i** using annexin-v and PI on Hela cell line. Treatment with 8 µM annexin-v and 16 µM PI for **8g**, and Treatment with 2.5 µM annexin-v and 1.5 µM PI for **8i**.

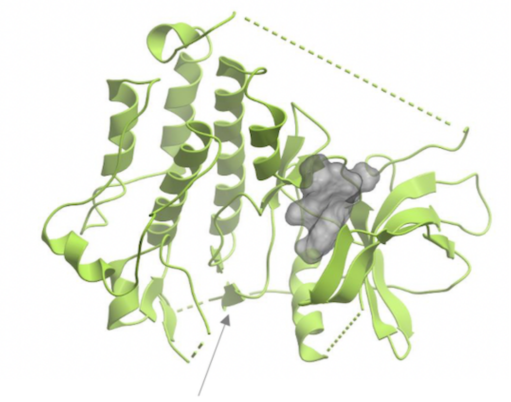

Figure S2. Ribbon representation of EGFR showing the binding pocket with a grey skin mesh (PDB ID: 4ZAU).

Table S1. Calculated binding properties of test compounds and sorafenib against EGFR.

Test compounds	Binding score	Hydrogen bonding energy (Kcal/mol)	Hydrophobic interaction energy (Kcal/mol)
**Sorafenib**	-38.13	-2.52	-7.12
**8d**	-32.33	-1.19	-7.97
**8g**	-16.46	-2.25	-7.62
**8i**	-12.98	-2.47	-7.50
**10c**	-23.74	-3.36	-7.35

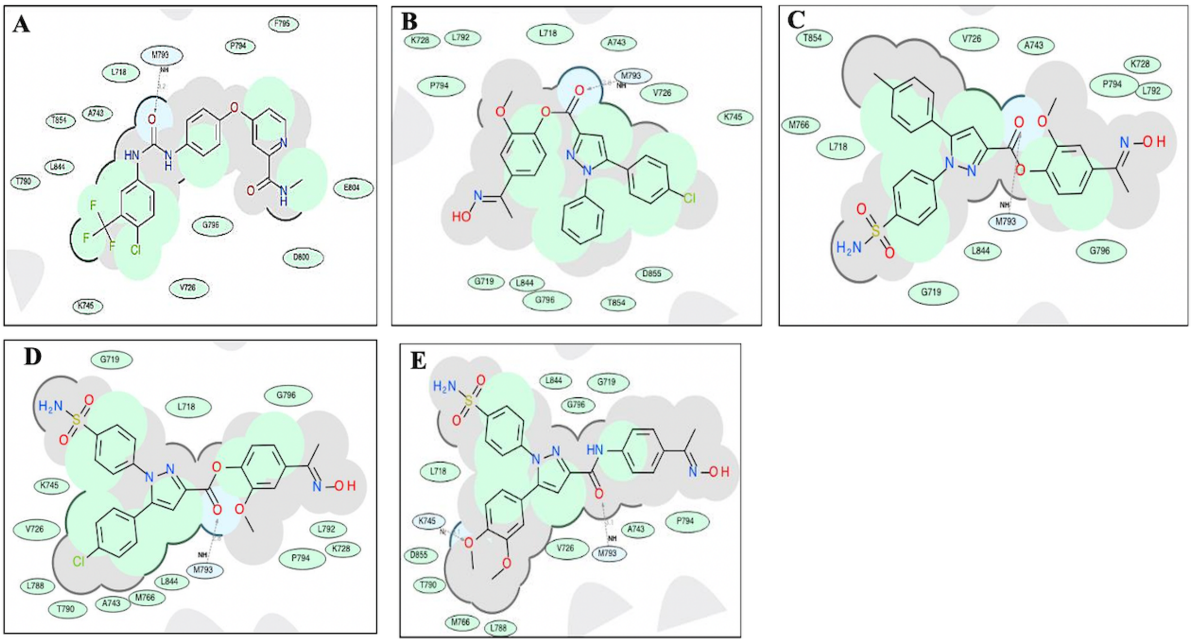

Figure S3. (A-E) Docking pose of sorafenib at the EGFR binding pocket.

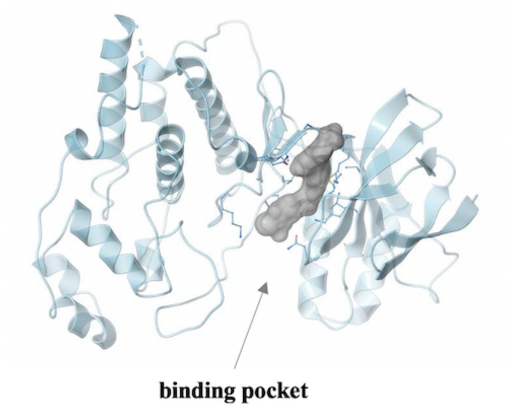

Figure S4. Ribbon representation of JNK-2 showing the binding pocket with a grey skin mesh (PDB ID: 3E7O) [15].

Table S2. Calculated binding properties of test compounds andsorafenib against JNK-2.

Test compounds	Binding score	Hydrogen bonding energy (Kcal/mol)	Hydrophobic interaction energy (Kcal/mol)	Van der Waals interaction energy (Kcal/mol)
**Sorafenib**	-21.48	-2.72	-8.46	-35.60
**8d**	-9.16	-0.99	-7.97	-33.70
**8i**	-12.00	-3.19	-7.66	-32.59

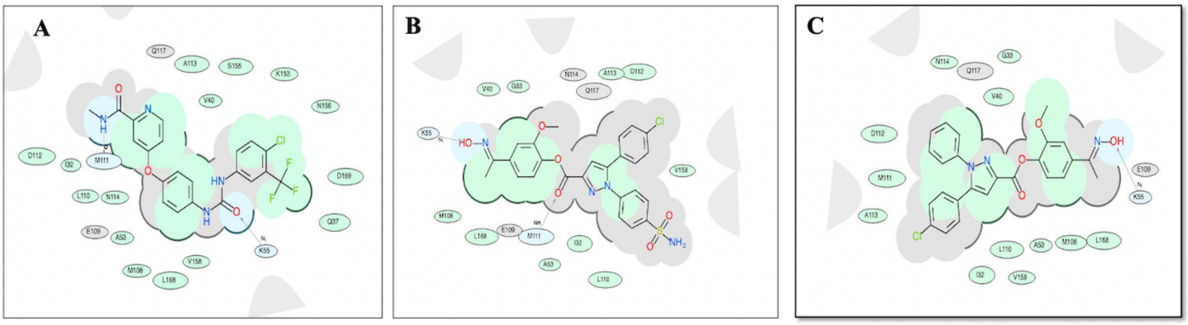

Figure S5. (A-C) Docking pose of sorafenib at the JNK-2 binding pocket.

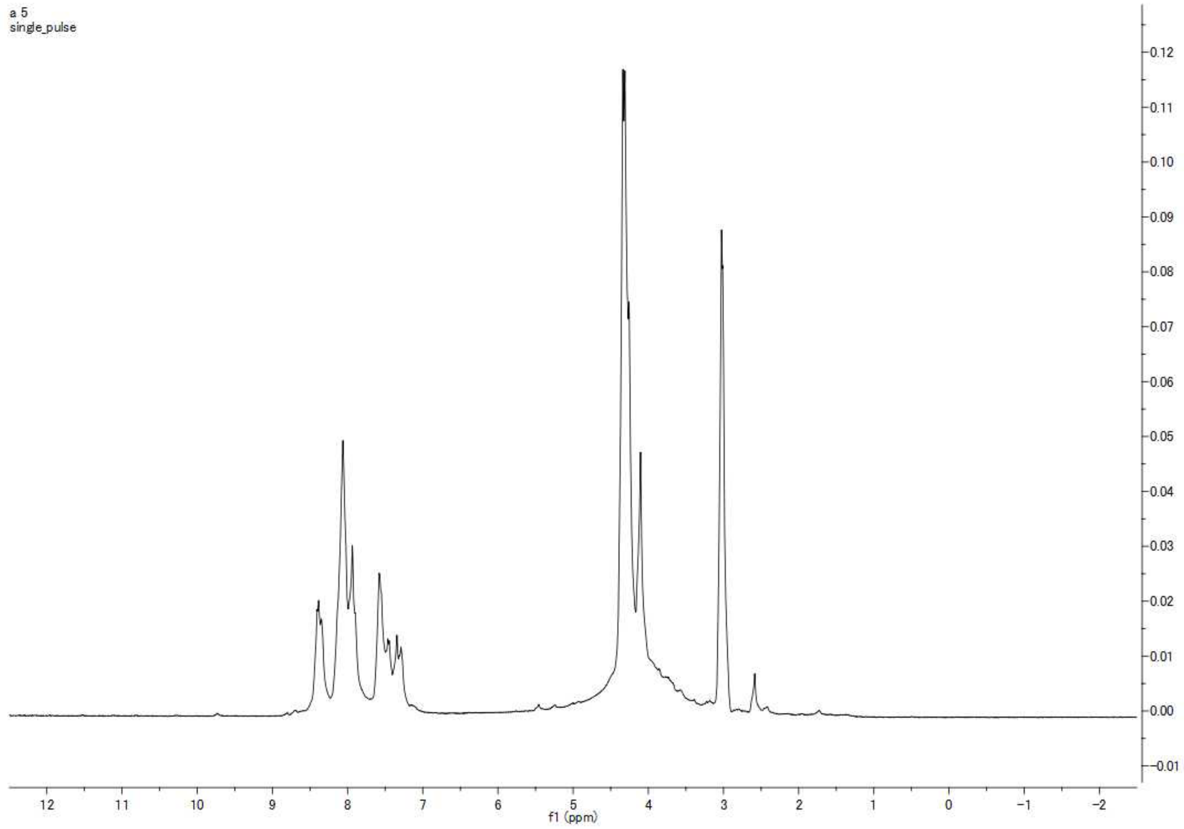

^{1}H-NMR of compound **6e** in DMSO-*d6*.

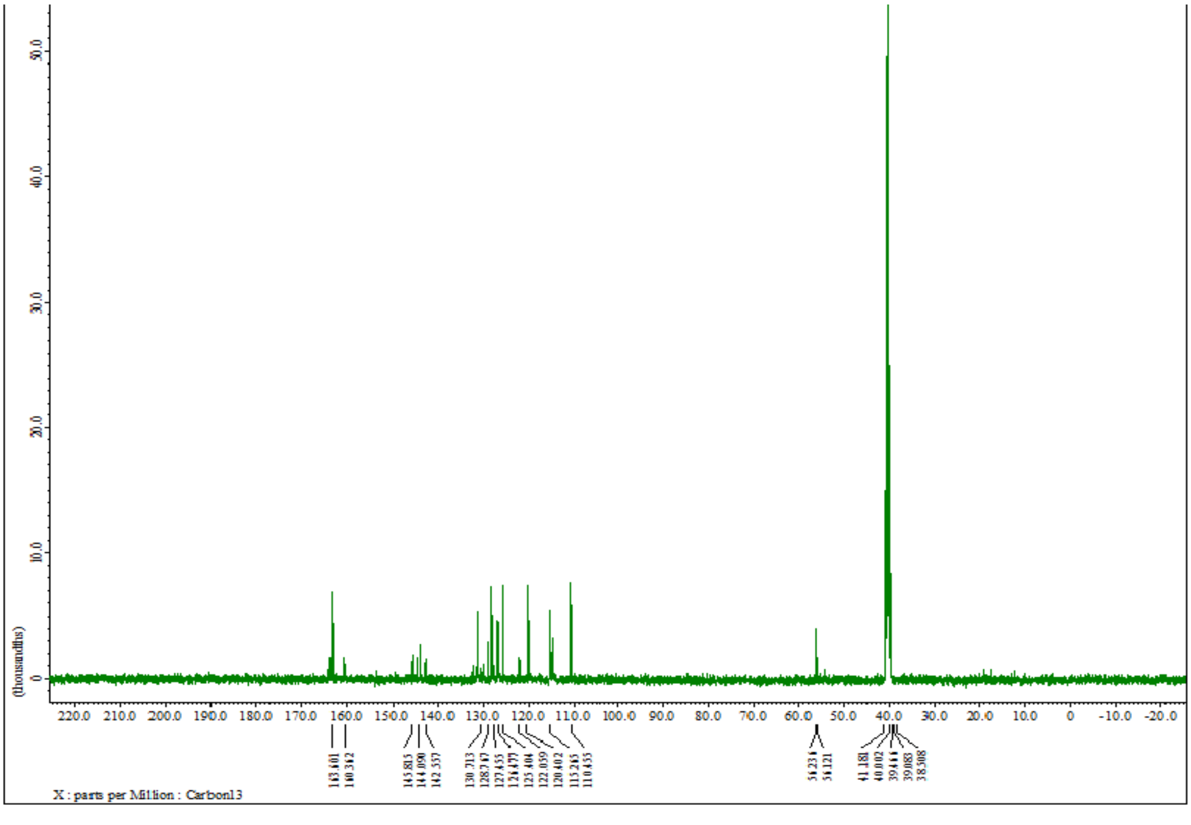

^{13}C-NMR of Compound **6e** in DMSO-*d6*.

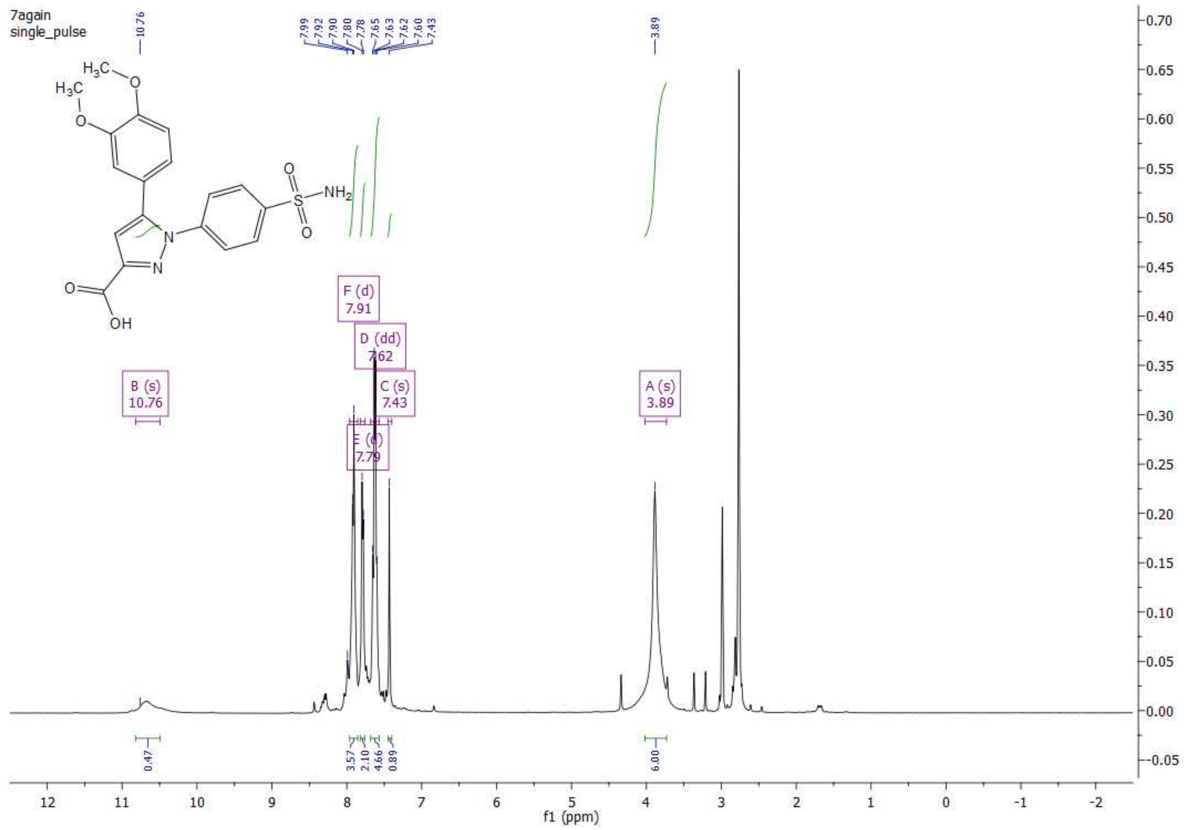

^{1}H-NMR of compound **6j** in DMSO-*d6*

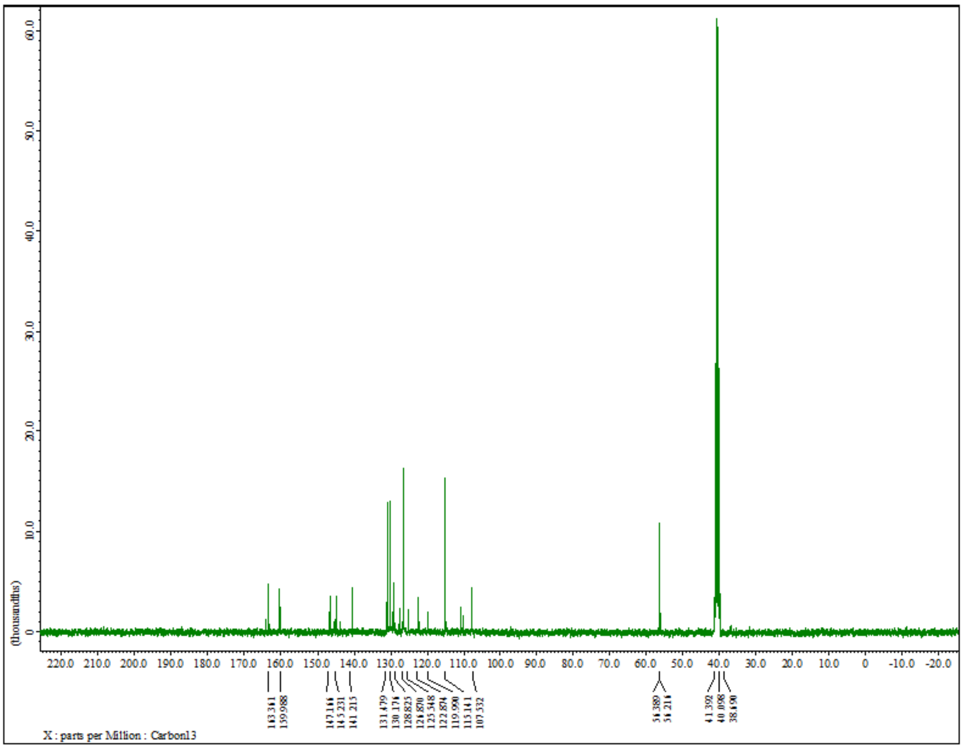

^{13}C-NMR of Compound **6j** in DMSO-*d6*.

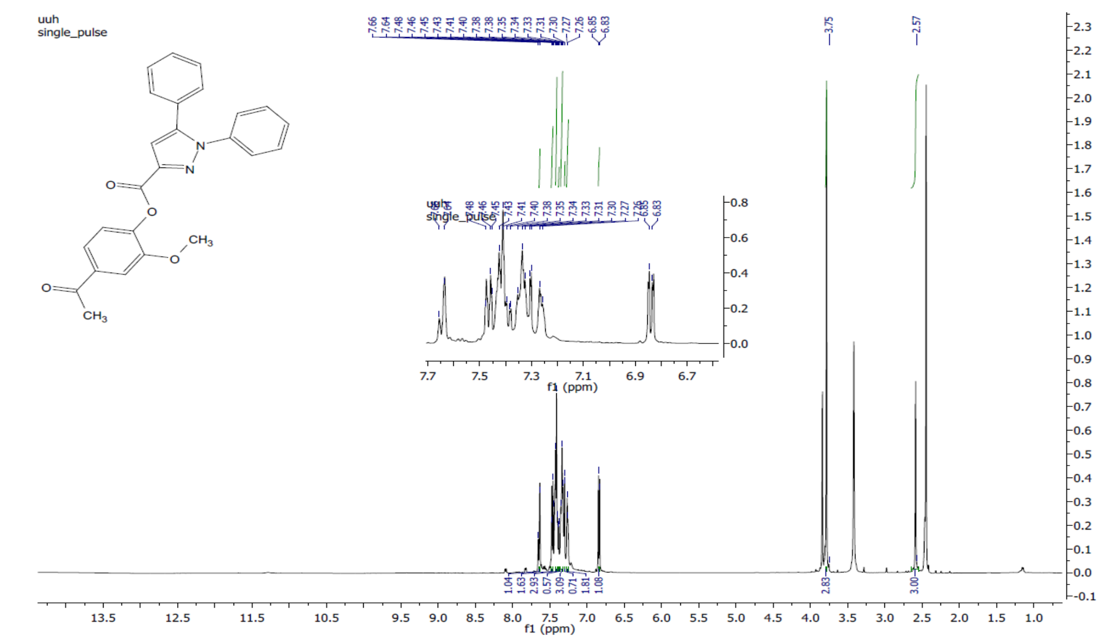

^{1}H-NMR of compound **7a** in DMSO-*d6*.

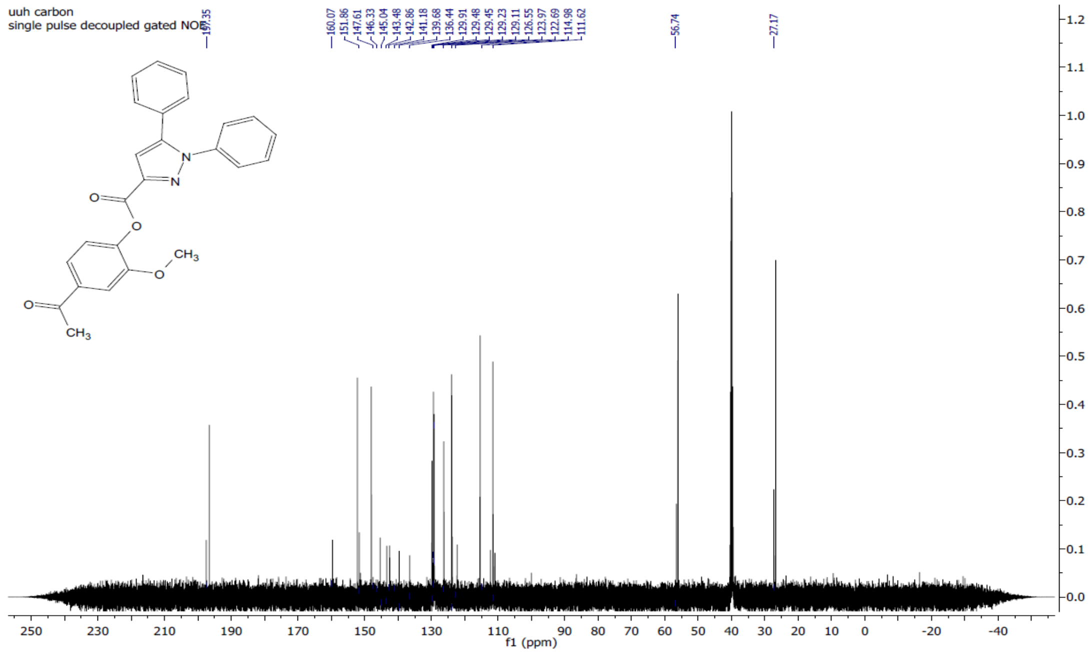

^{13}C-NMR of Compound **7a** in DMSO-*d6*.

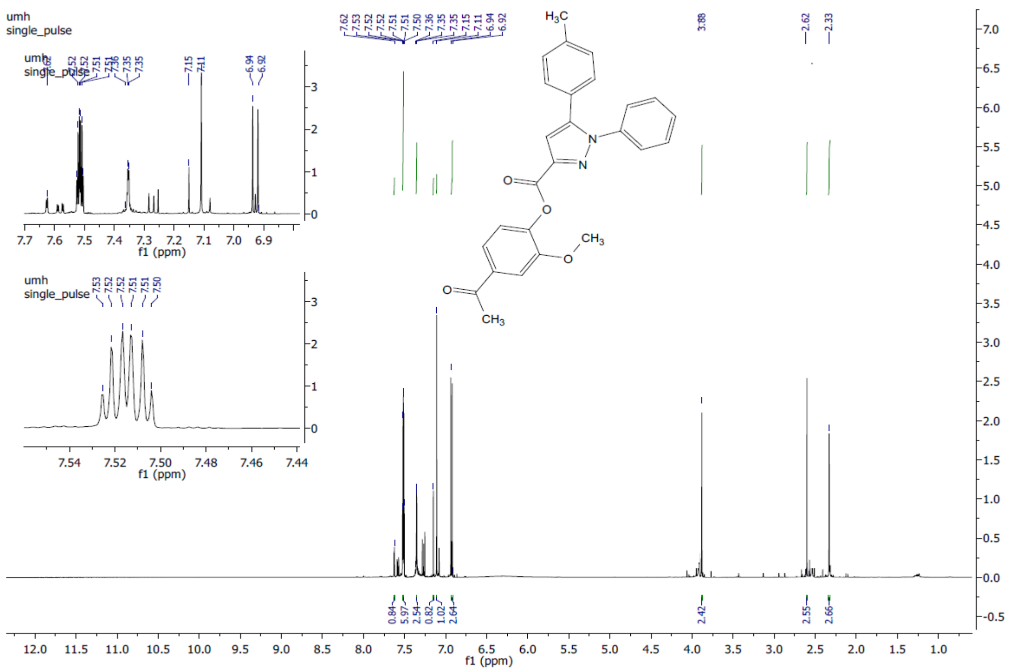

^{1}H-NMR of compound **7b** in $CDCl_3$.

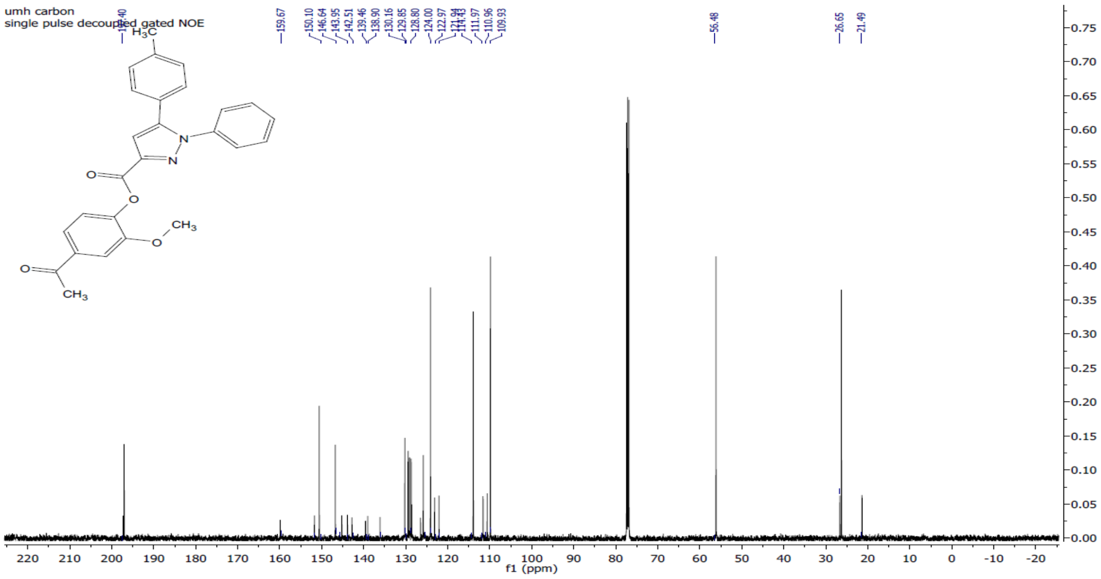

^{13}C-NMR of Compound **7b** in $CDCl_3$.

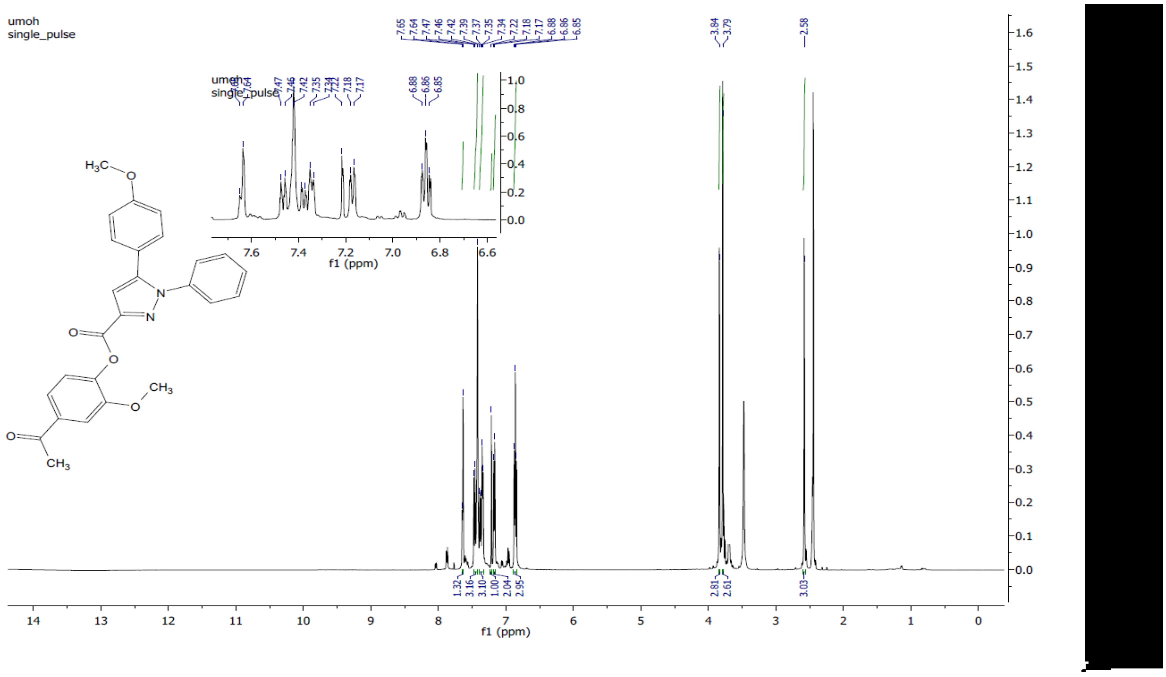

^{1}H-NMR of compound **7c** in DMSO-*d6*.

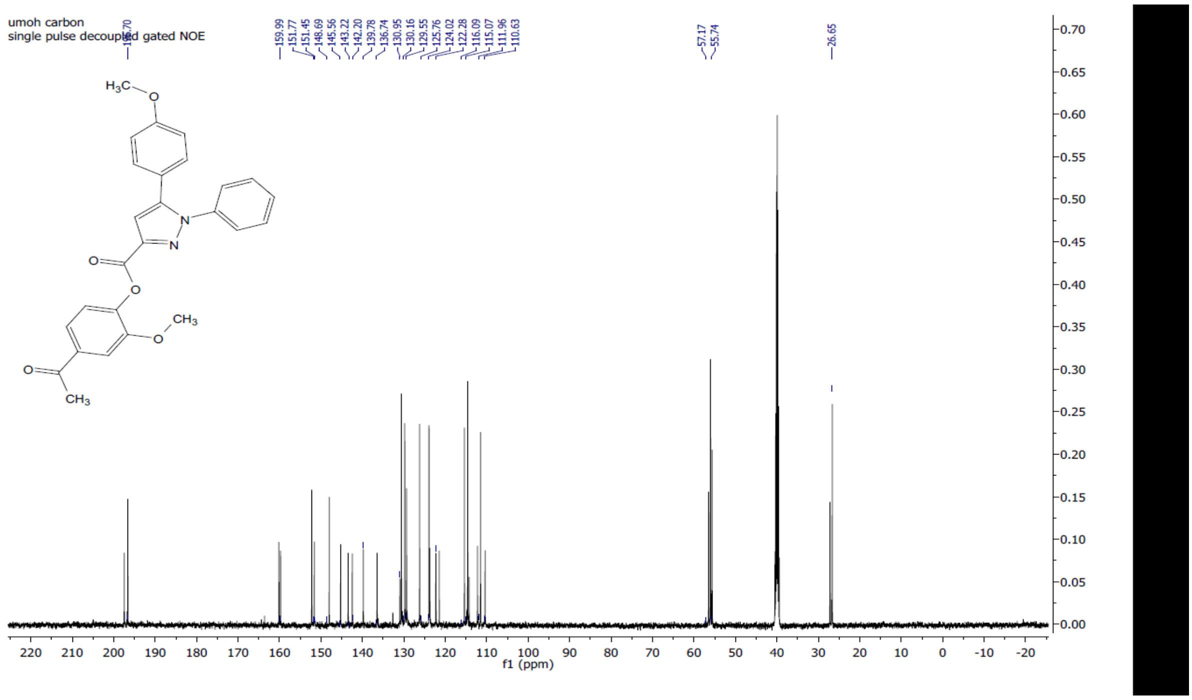

^{13}C-NMR of Compound **7c** in DMSO-*d6*.

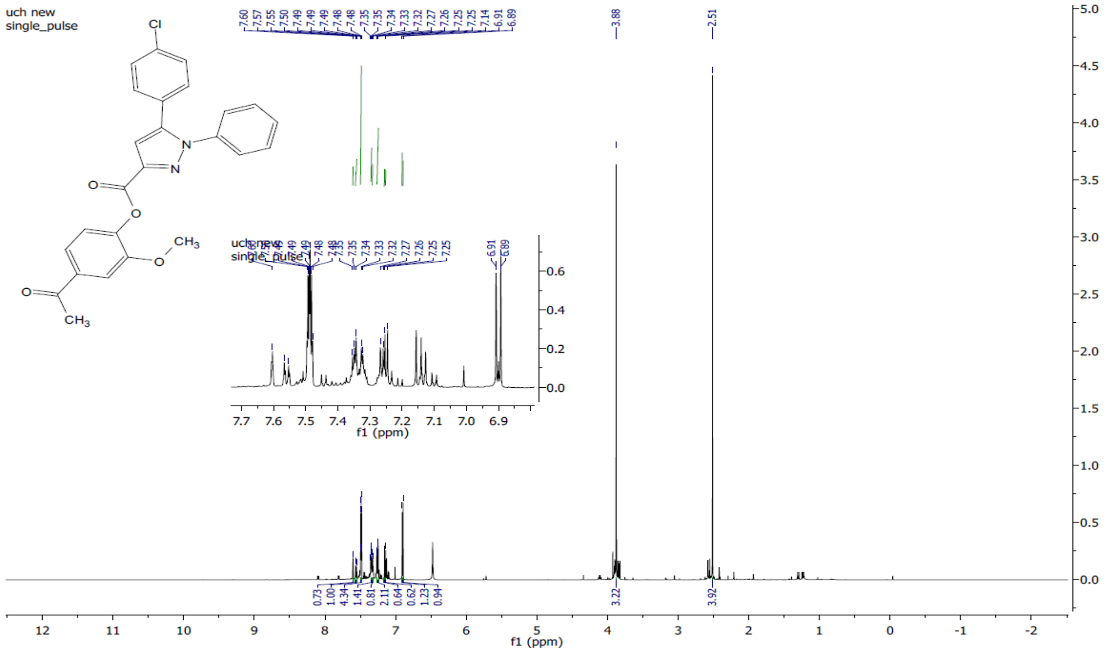

^{1}H-NMR of compound **7d** in $CDCl_3$.

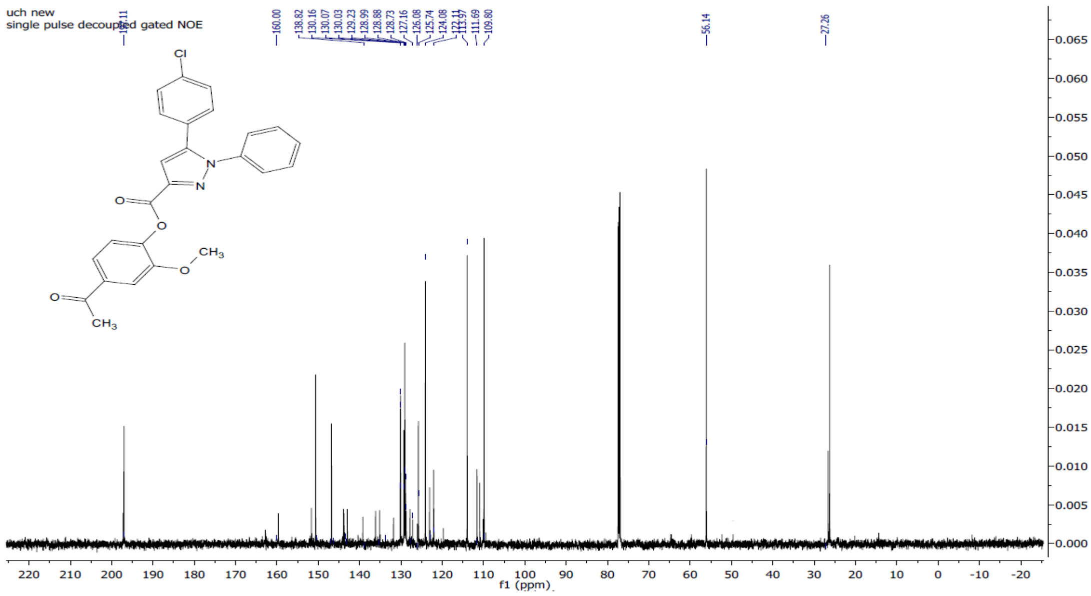

^{13}C-NMR of Compound **7d** in $CDCl_3$.

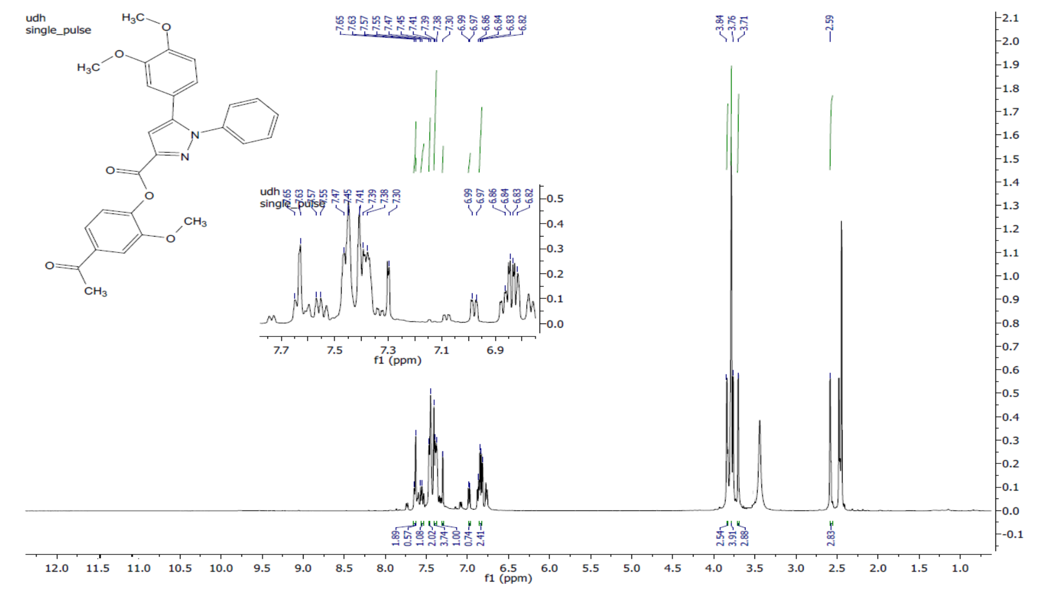

^{1}H-NMR of compound **7e** in DMSO-*d6*.

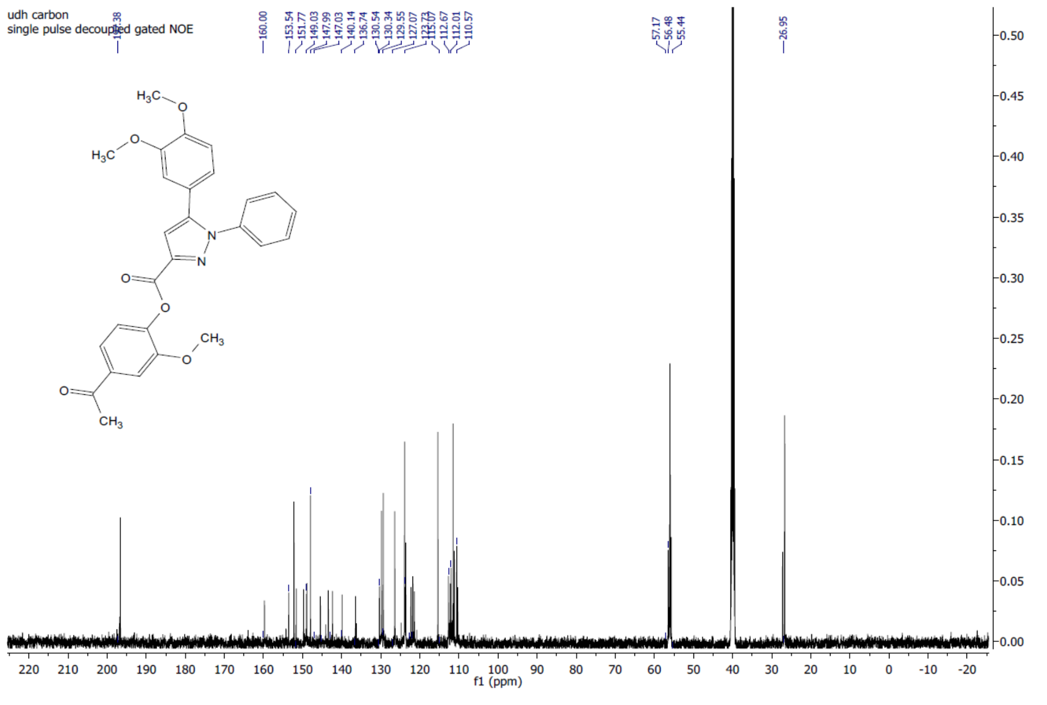

^{13}C-NMR of Compound **7e** in DMSO-*d6*.

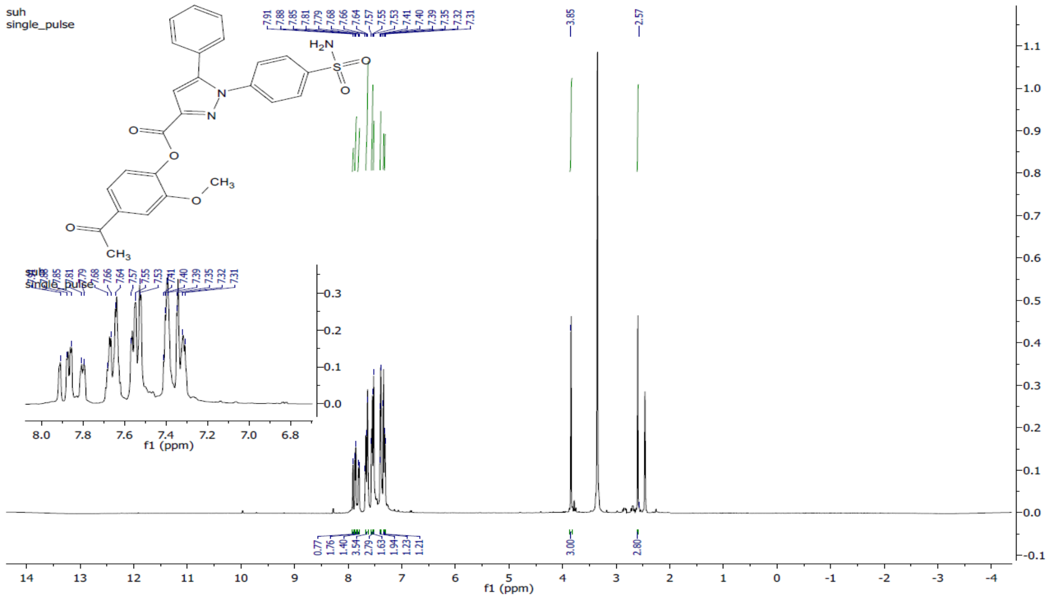

^{1}H-NMR of compound **7f** in DMSO-*d6*.

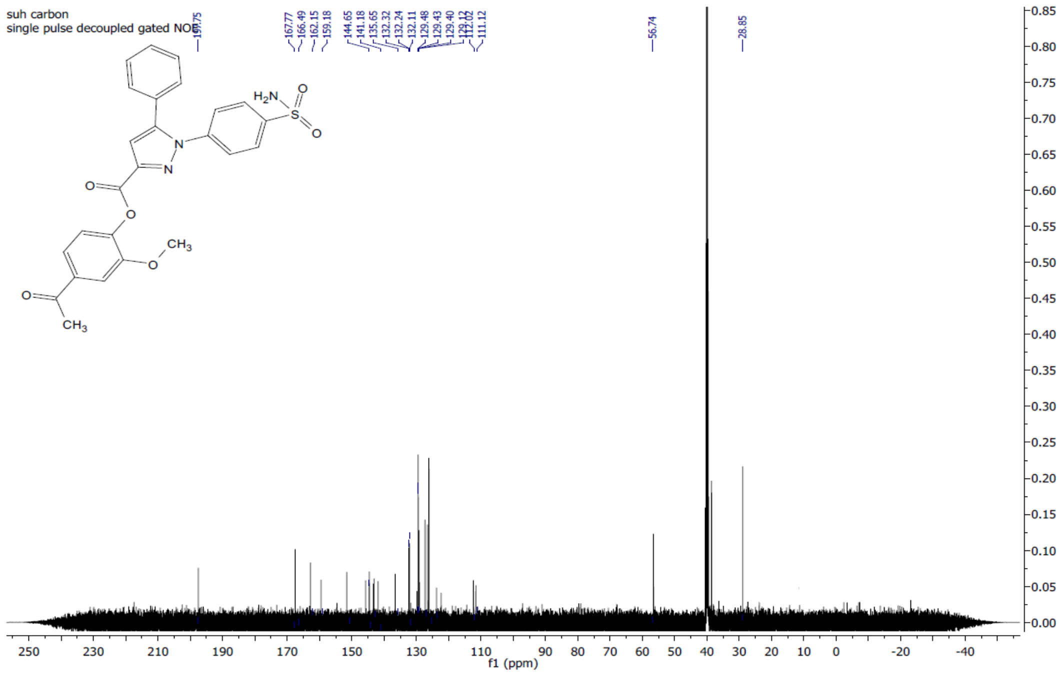

^{13}C-NMR of Compound **7f** in DMSO-*d6*.

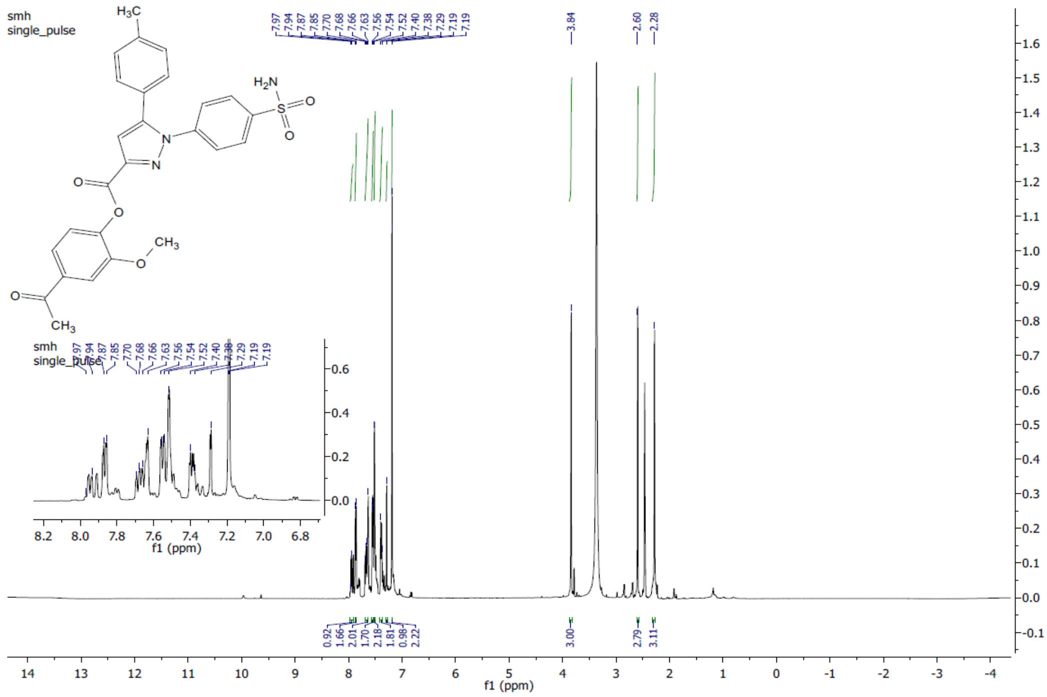

^{1}H-NMR of compound **7g** in DMSO-*d6*.

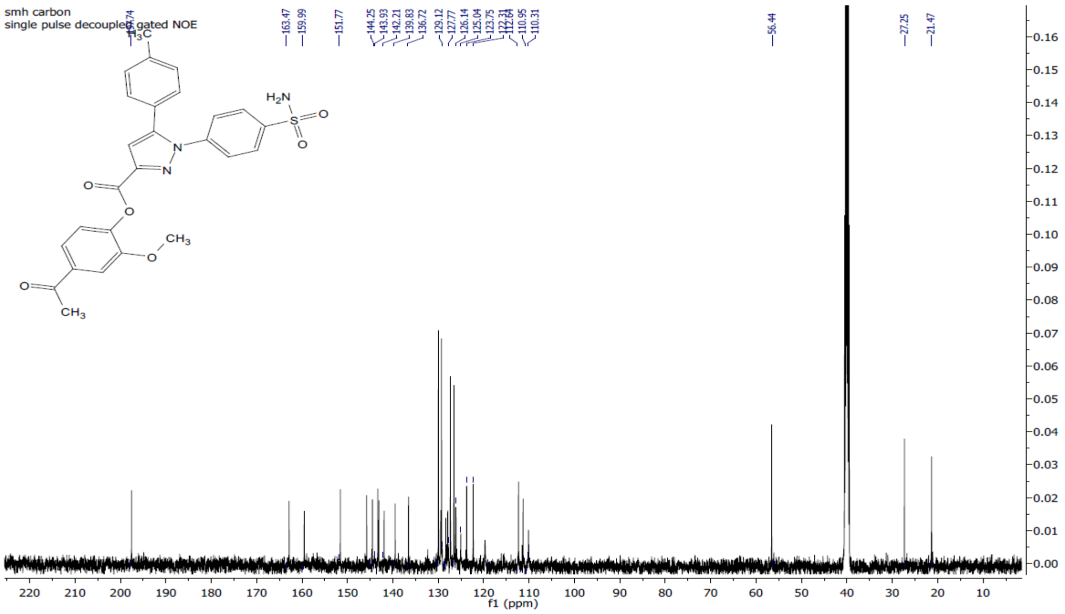

^{13}C-NMR of Compound **7g** in DMSO-*d6*.

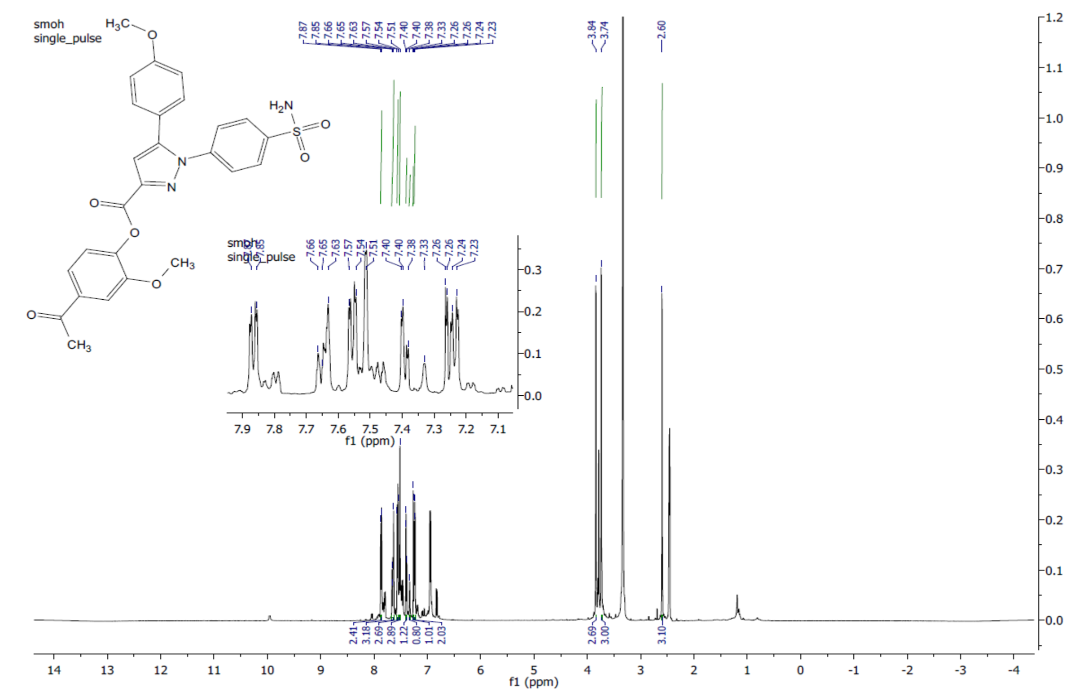

^{1}H-NMR of compound **7h** in DMSO-*d6*.

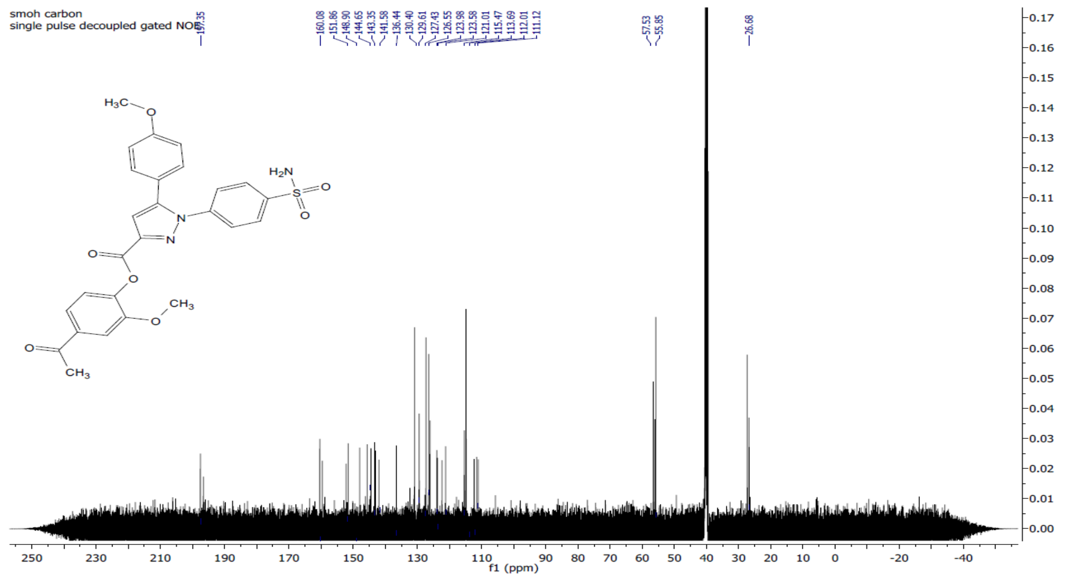

^{13}C-NMR of Compound **7h** in DMSO-*d6*.

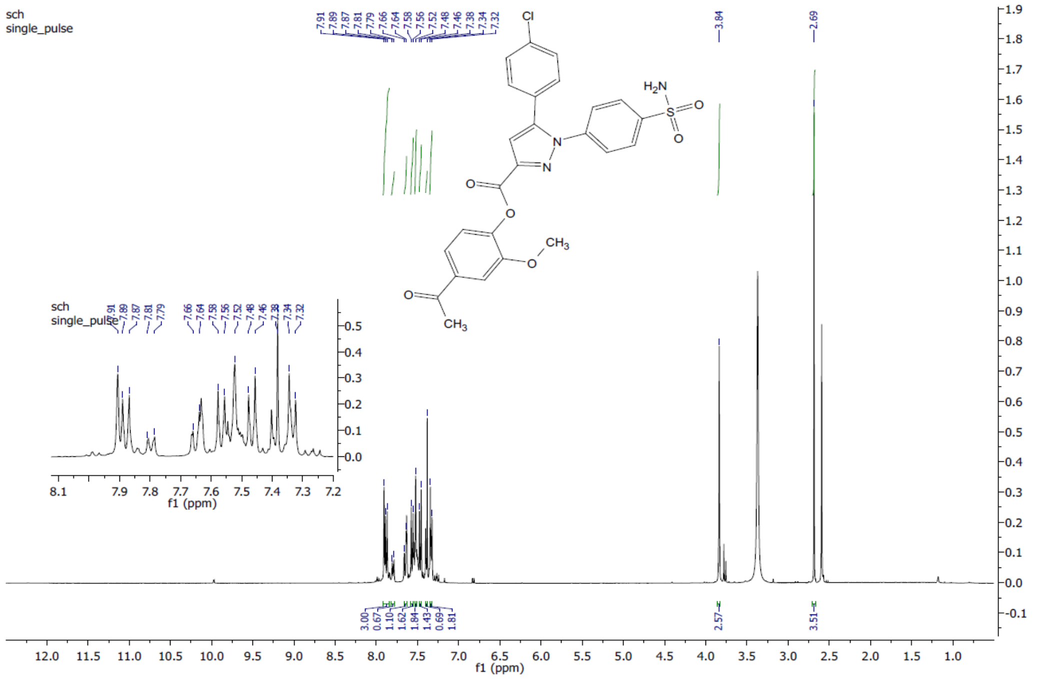

^{1}H-NMR of compound **7i** in DMSO-*d6*.

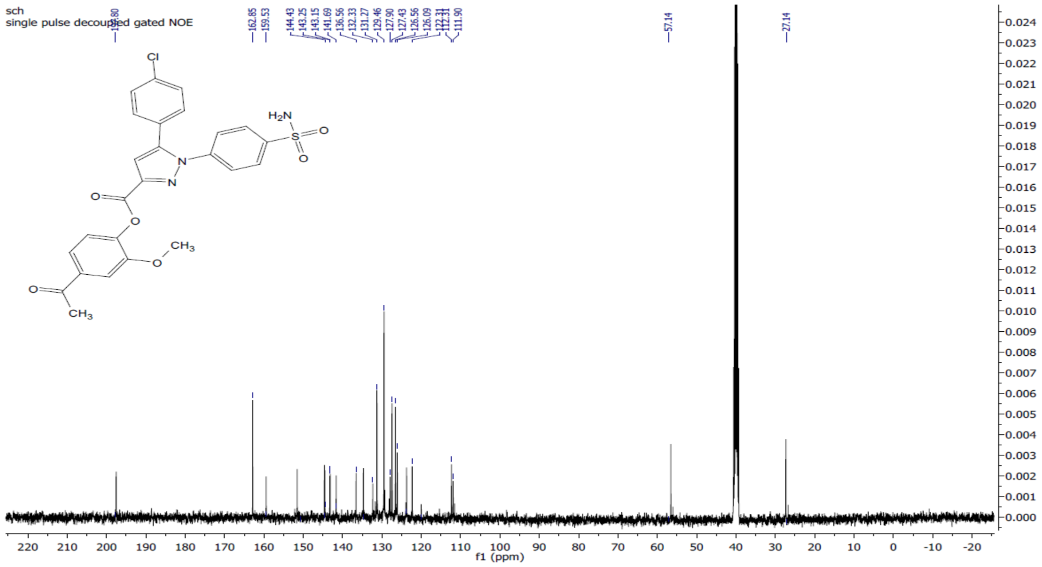

^{13}C-NMR of Compound **7i** in DMSO-*d6*.

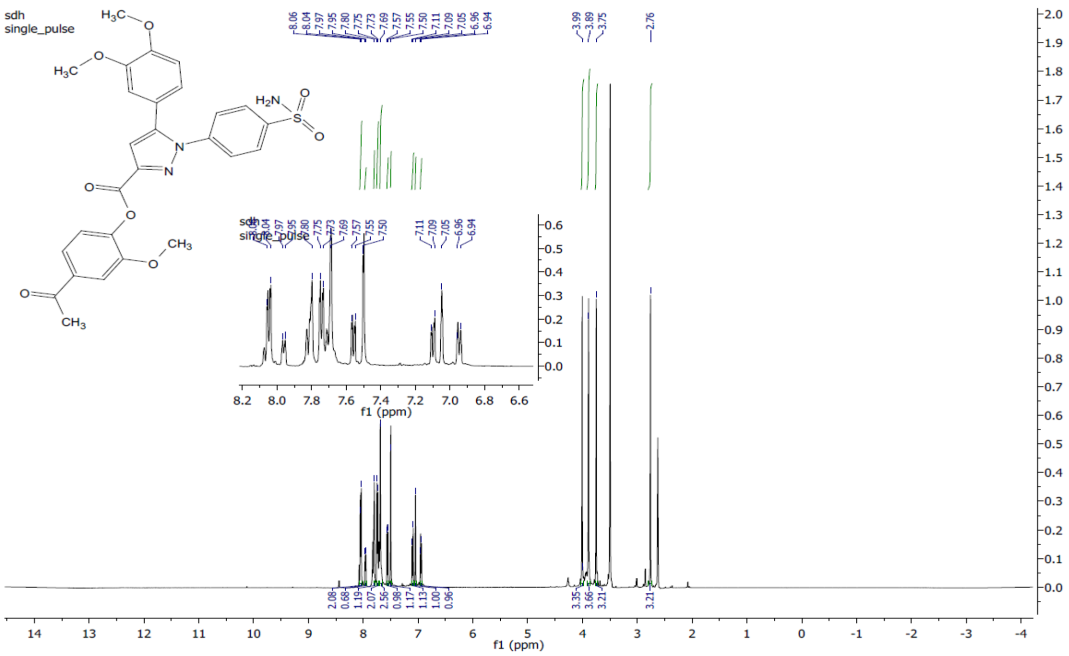

^{1}H-NMR of compound **7j** in DMSO-*d6*.

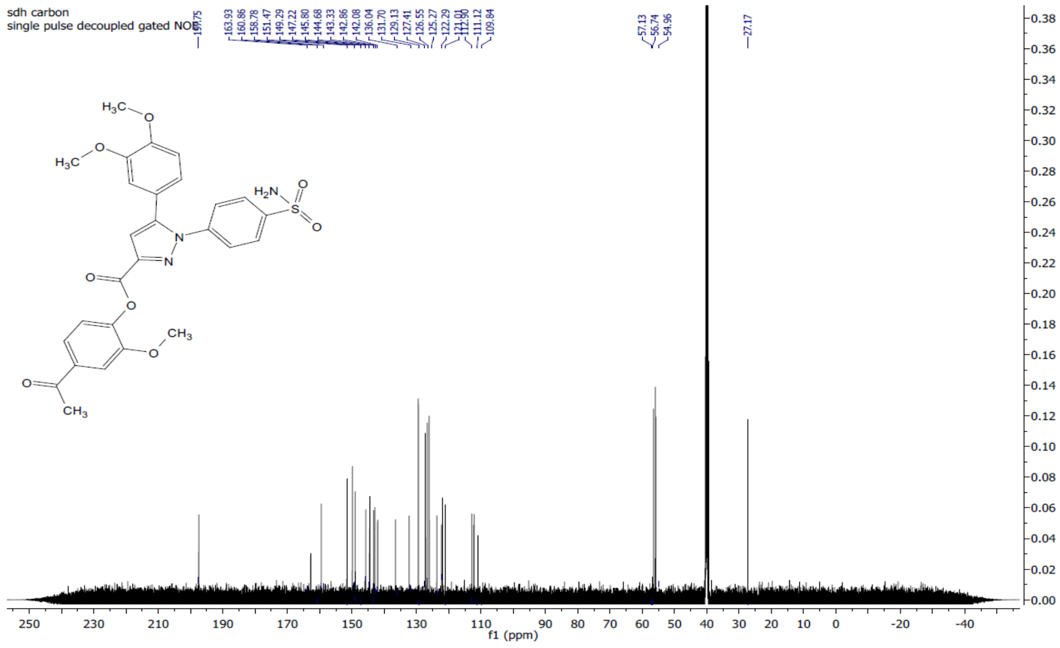

^{13}C-NMR of Compound **7j** in DMSO-*d6*.

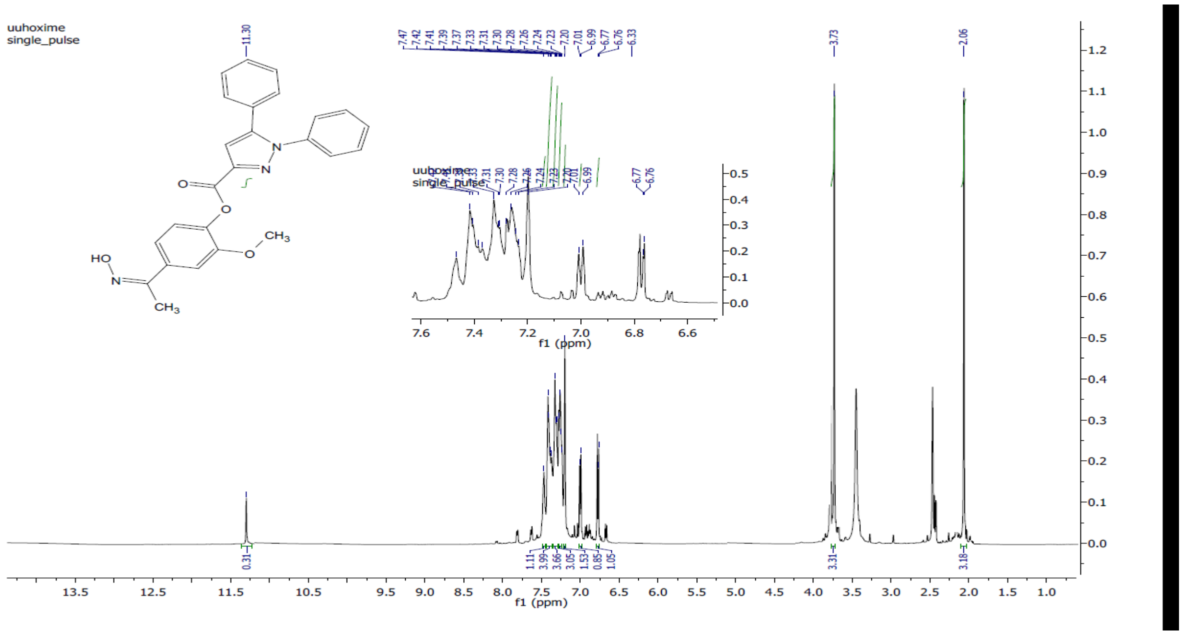

^{1}H-NMR of compound **8a** in DMSO-*d6*

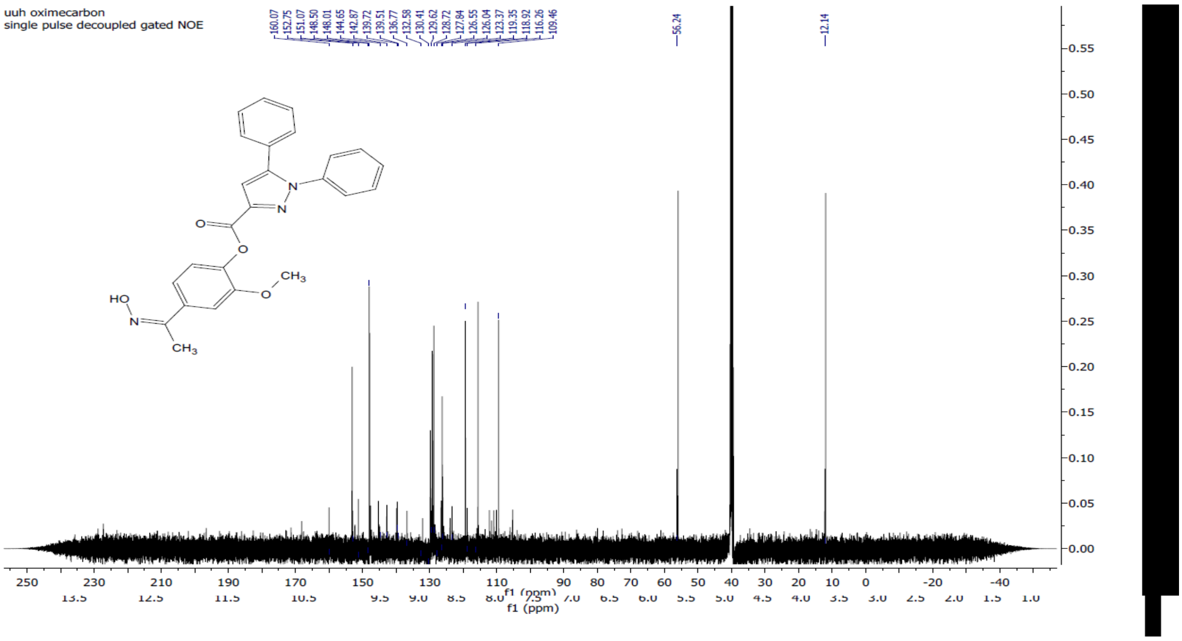

^{13}C-NMR of Compound **8a** in DMSO-*d6*.

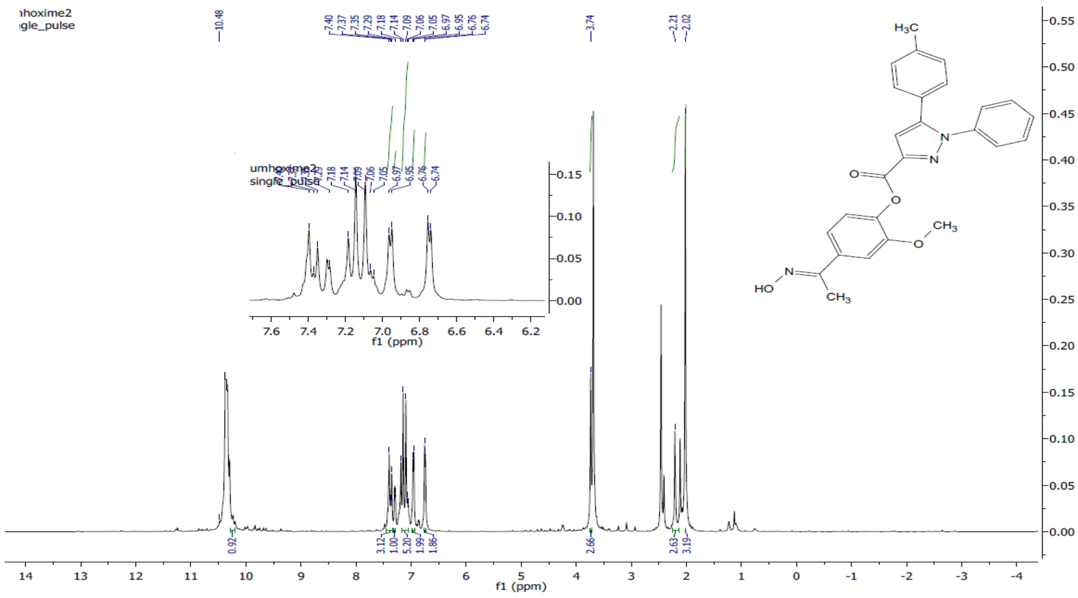

^{1}H-NMR of compound **8b** in DMSO-*d6*.

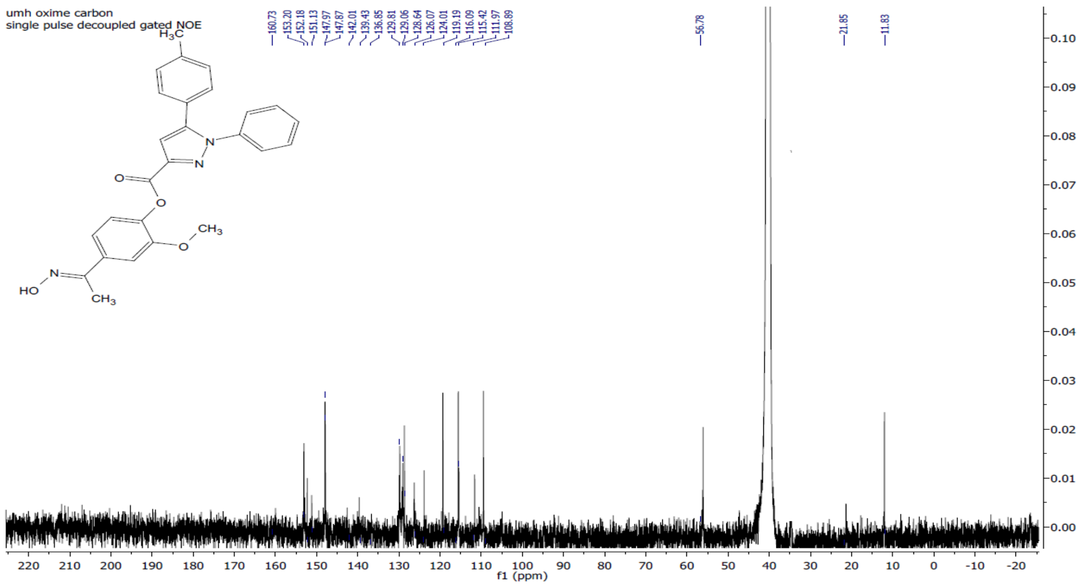

^{13}C-NMR of Compound **8b** in DMSO-*d6*.

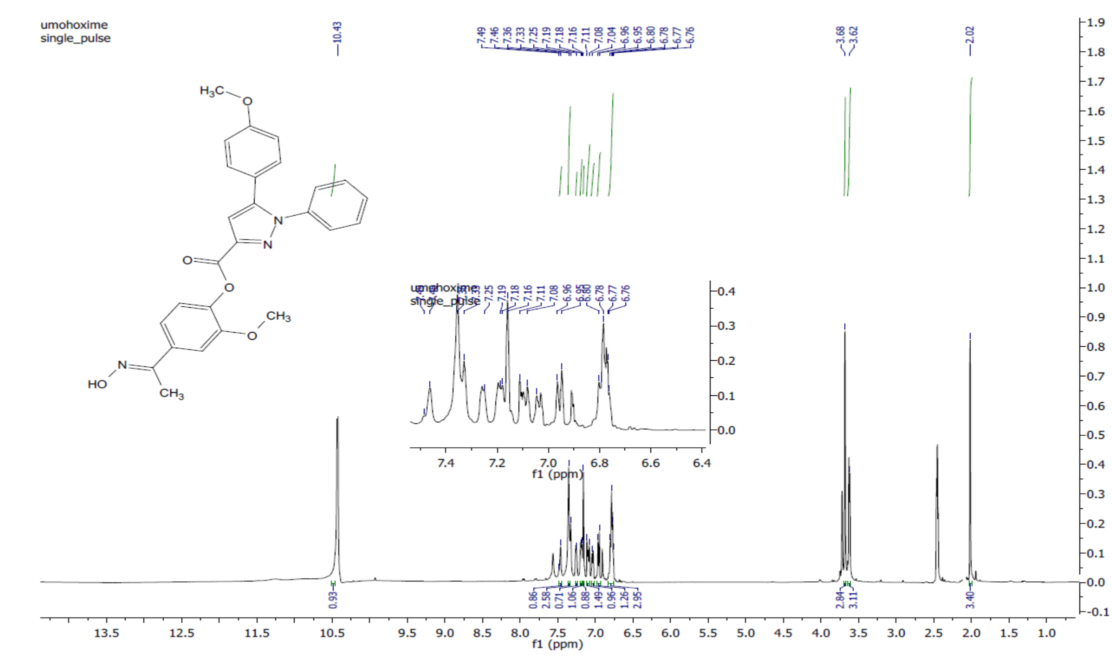

^{1}H-NMR of compound **8c** in DMSO-*d6*.

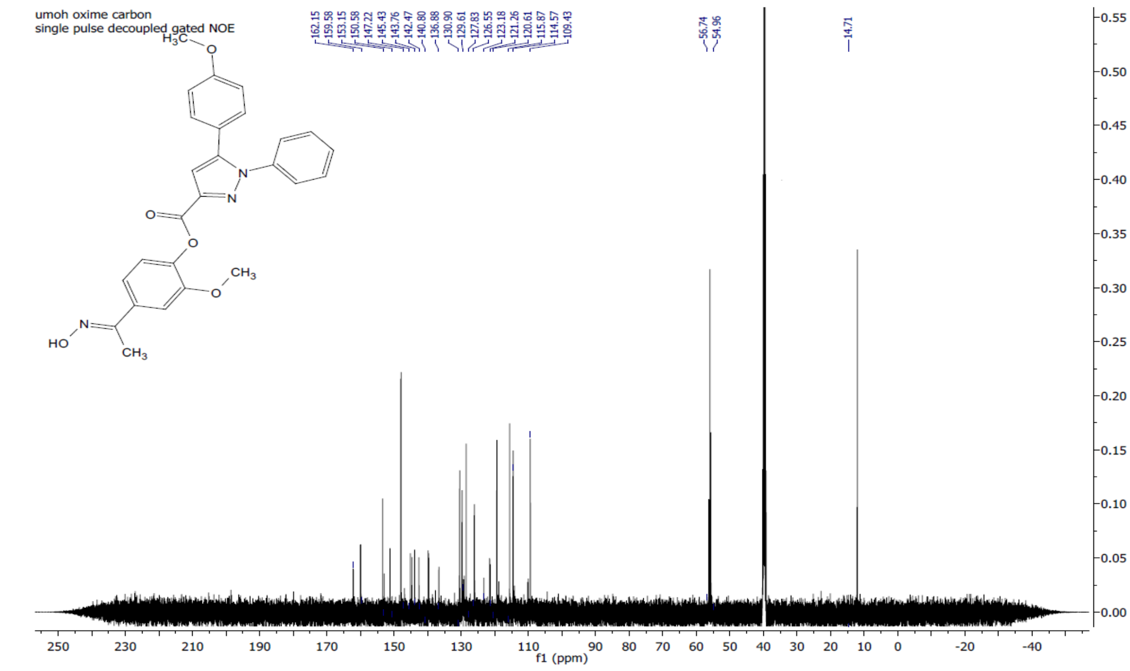

^{13}C-NMR of Compound **8c** in DMSO-*d6*.

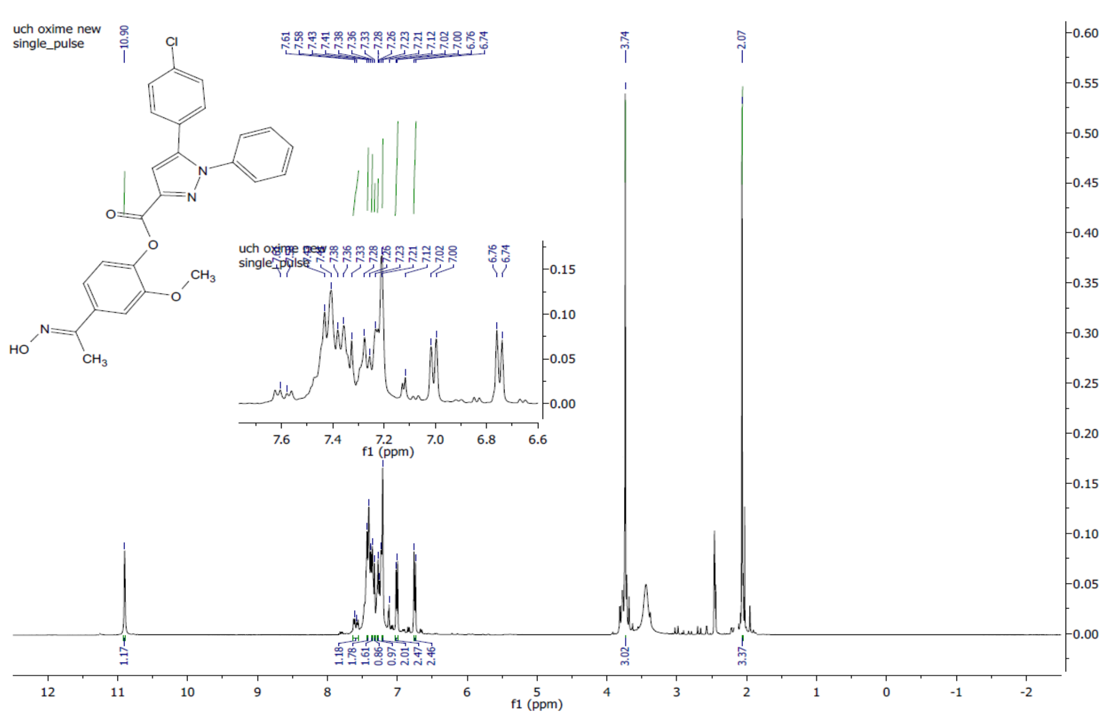

^{1}H-NMR of compound **8d** in DMSO-*d6*.

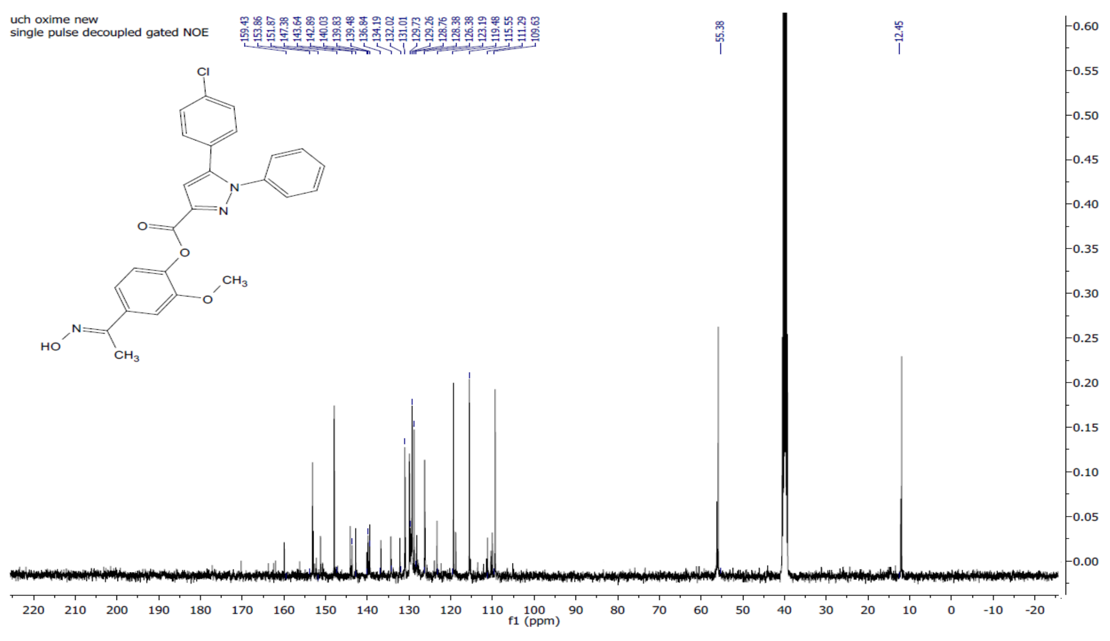

^{13}C-NMR of Compound **8d** in DMSO-*d6*.

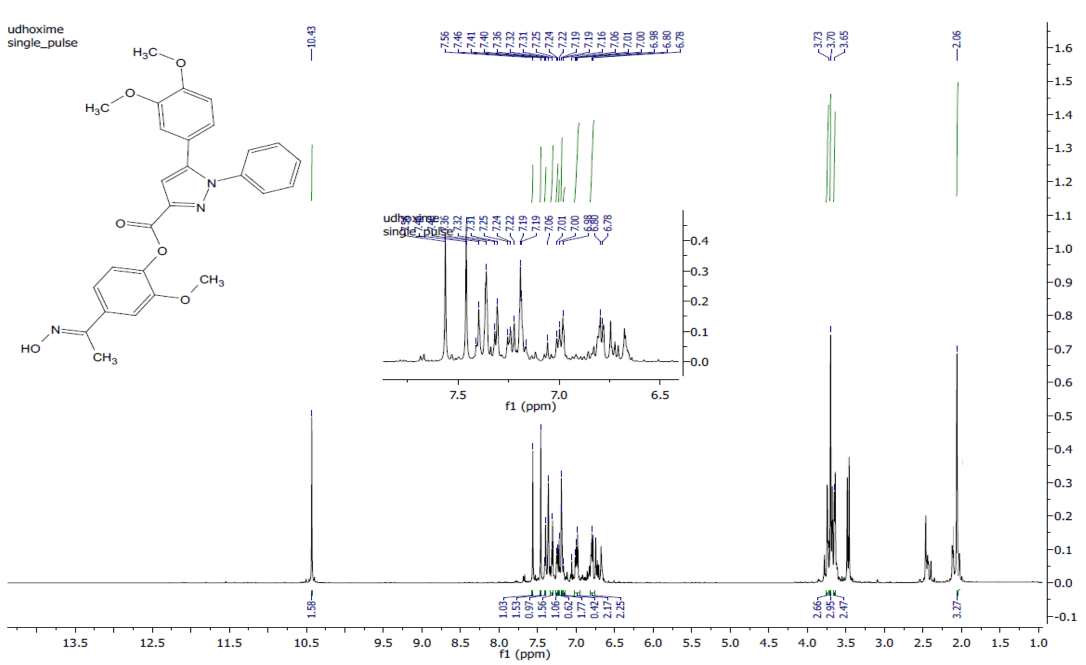

Fig. 49: ^{1}H-NMR of compound **8e** in DMSO-*d6.*

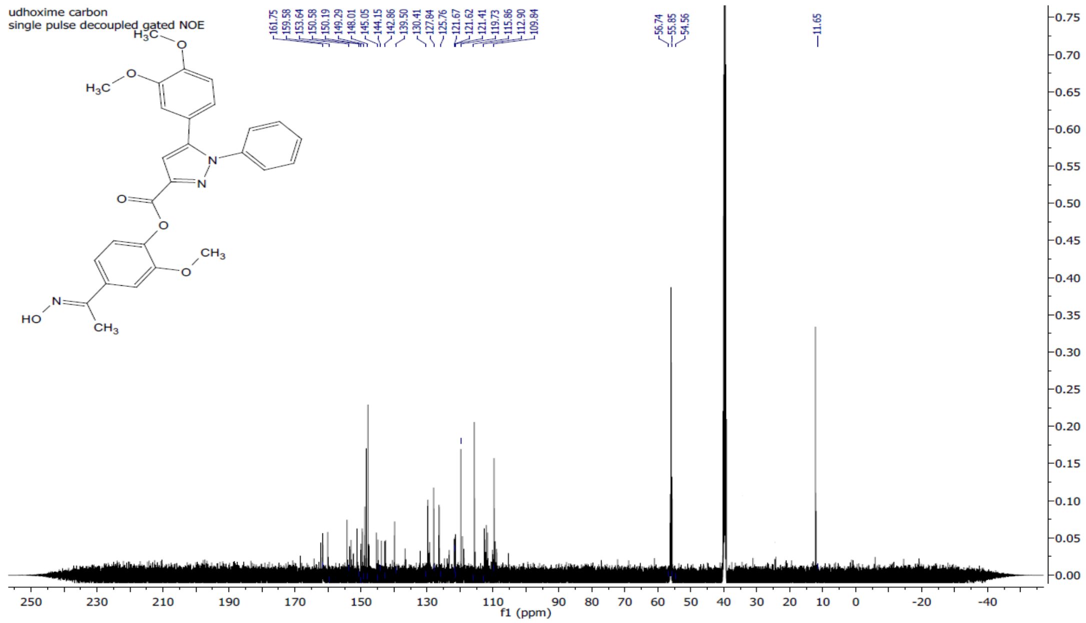

^{13}C-NMR of Compound **8e** in DMSO-*d6*.

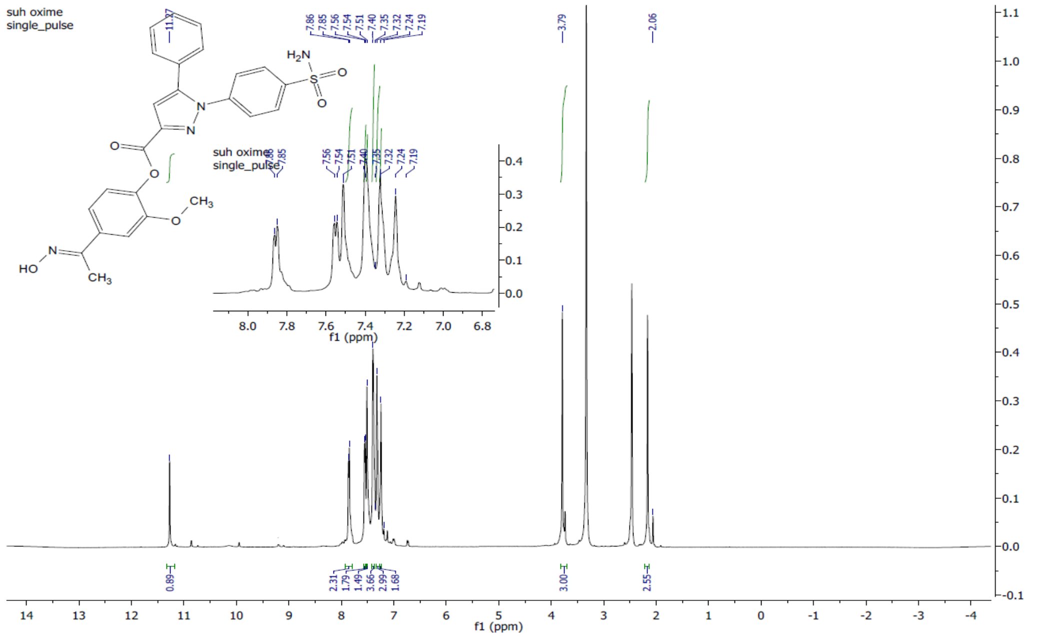

Fig. 52: ^{1}H-NMR of compound **8f** in DMSO-*d6*.

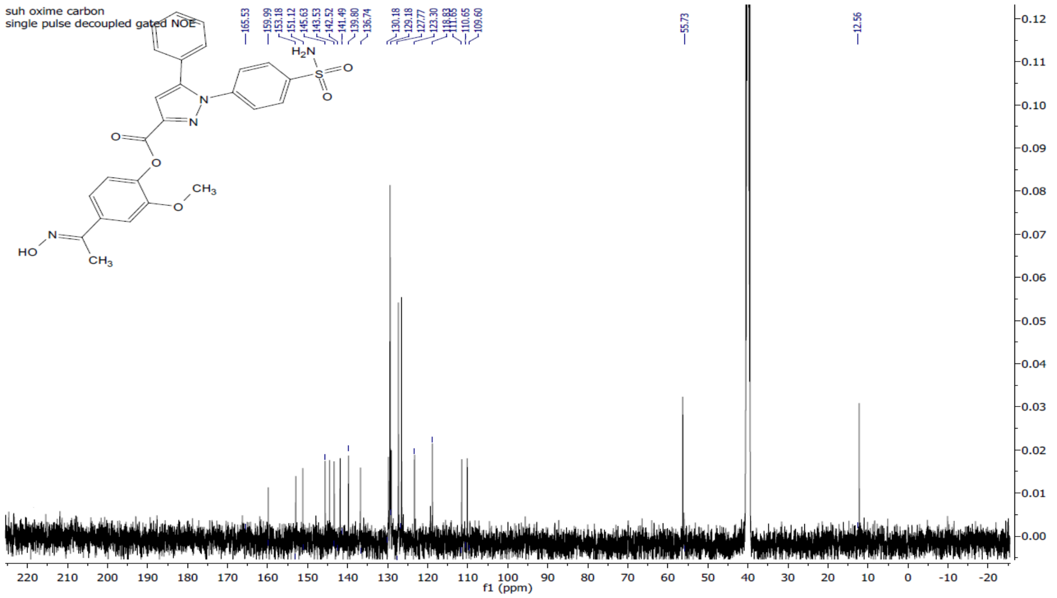

^{13}C-NMR of Compound **8f** in DMSO-*d6*.

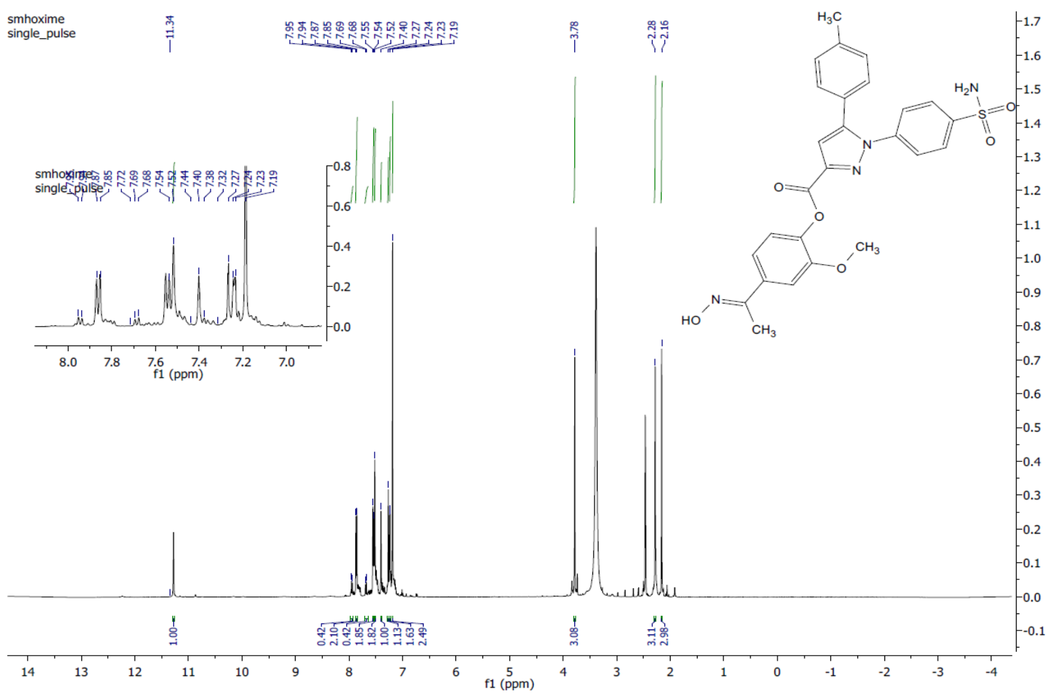

^{1}H-NMR of compound **8g** in DMSO-*d6*.

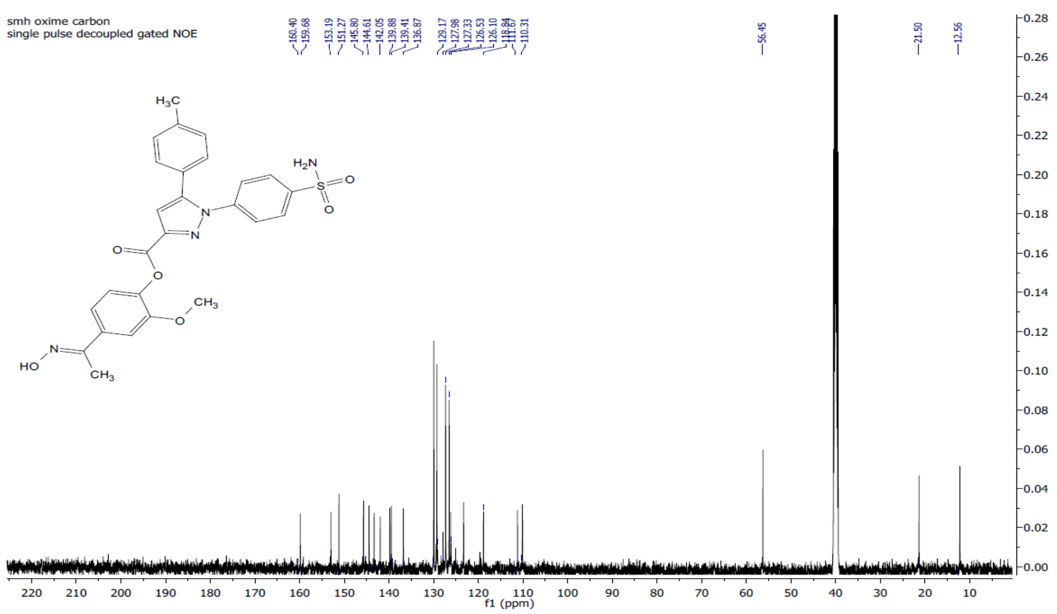

^{13}C-NMR of Compound **8g** in DMSO-*d6*.

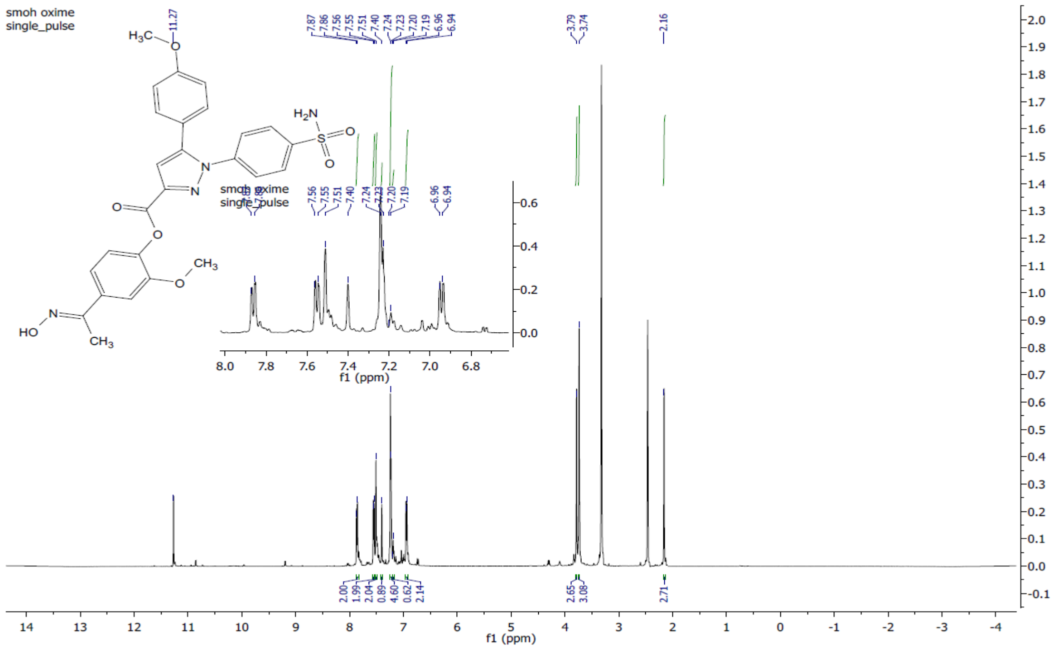

^{1}H-NMR of compound **8h** in DMSO-*d6*.

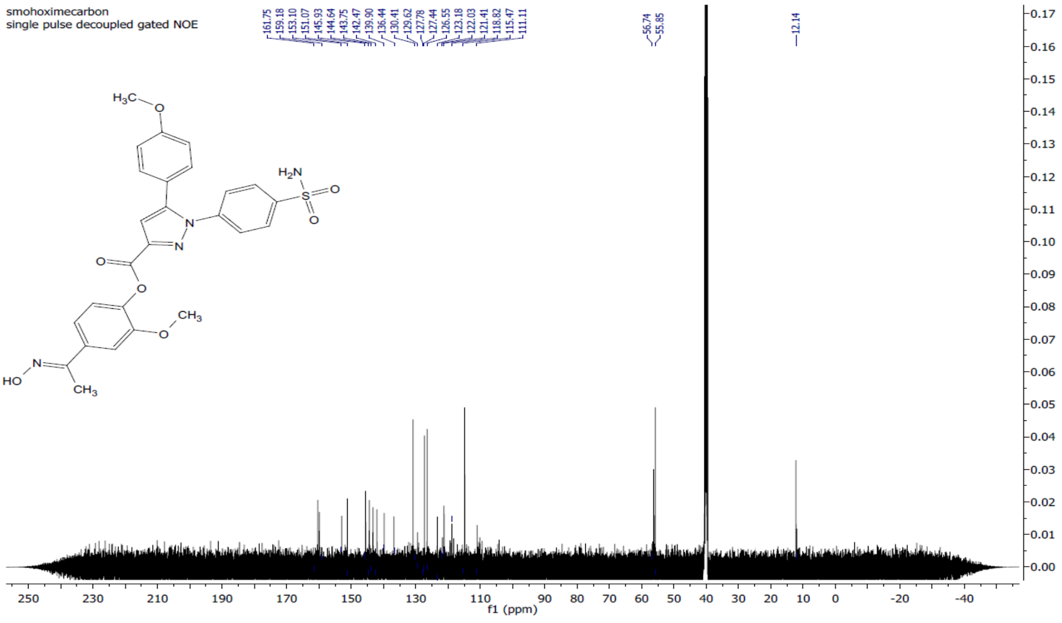

^{13}C-NMR of Compound **8h** in DMSO-*d6*.

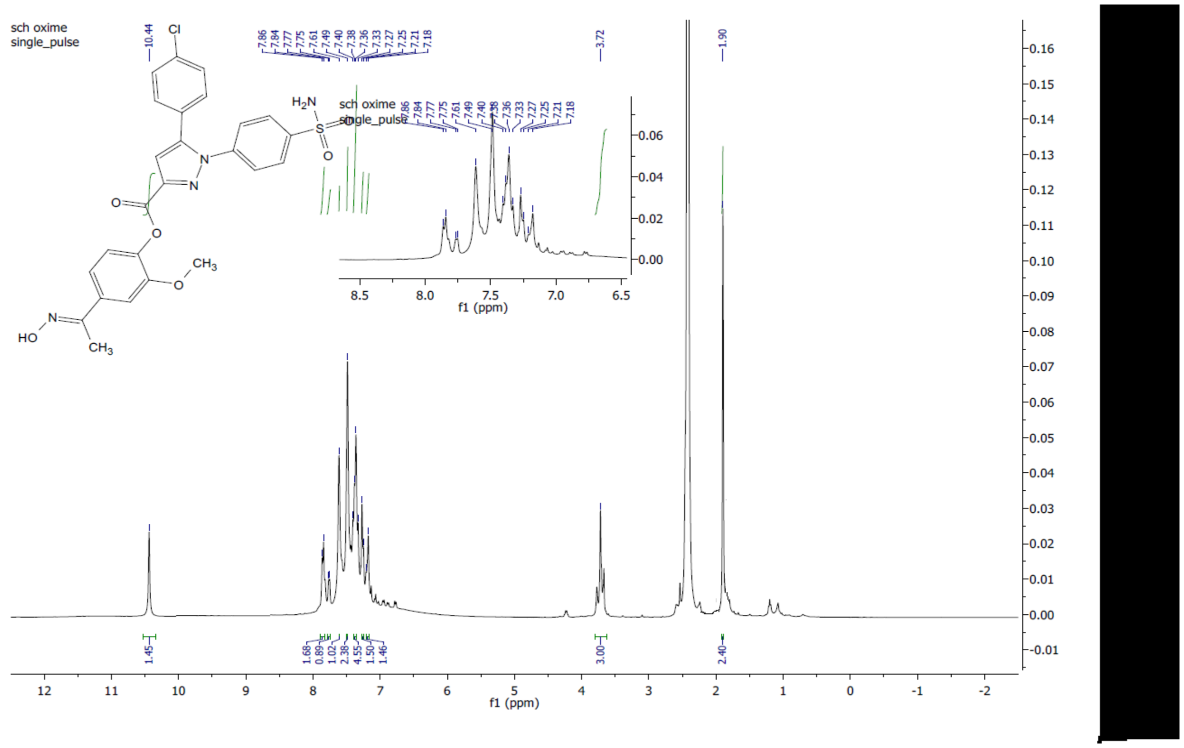

^{1}H-NMR of compound **8i** in DMSO-*d6*.

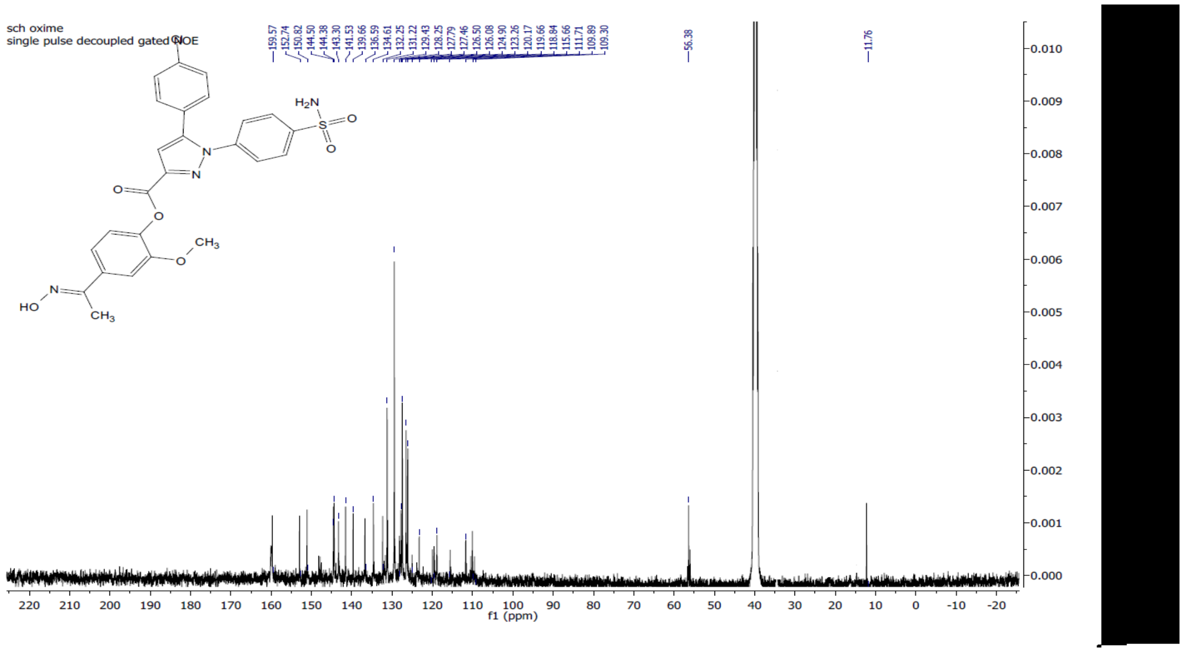

^{13}C-NMR of Compound **8i** in DMSO-*d6*.

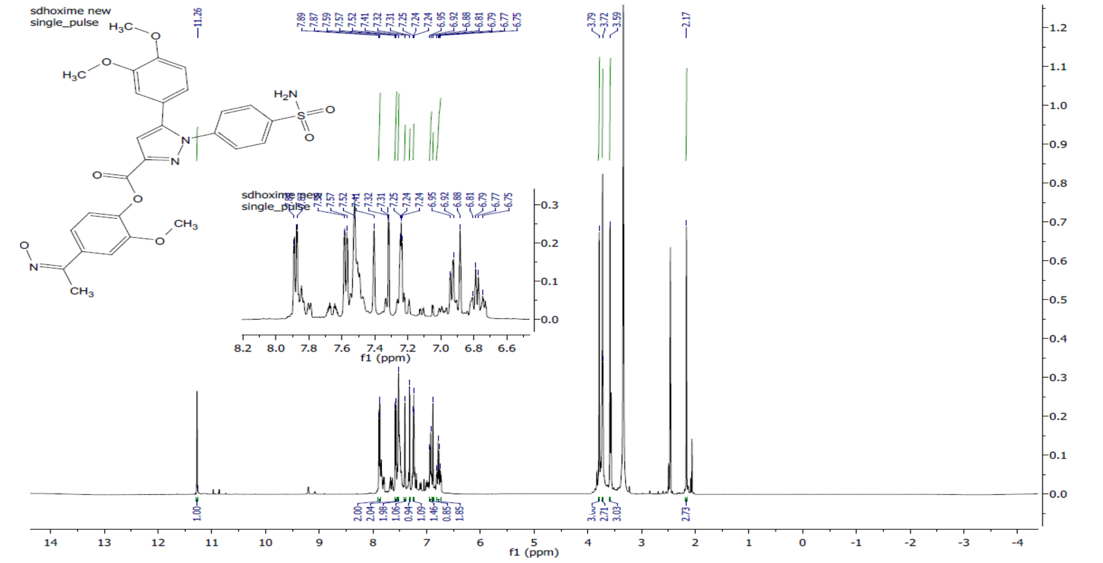

^{1}H-NMR of compound **8j** in DMSO-*d6*.

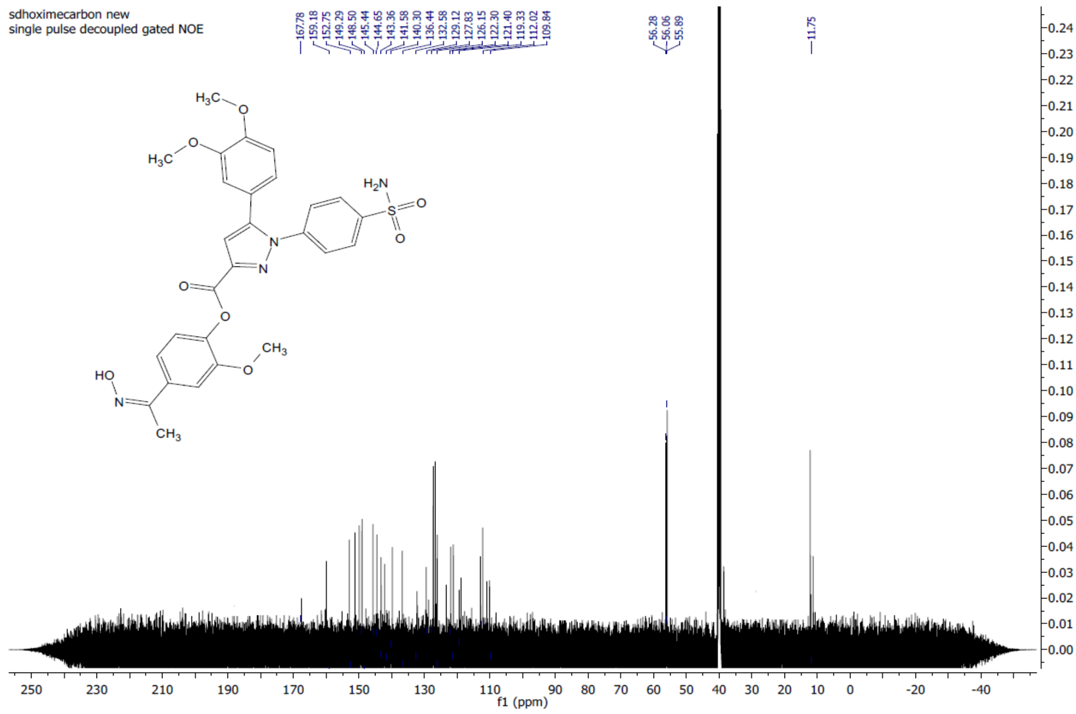

^{13}C-NMR of Compound **8j** in DMSO-*d6*.

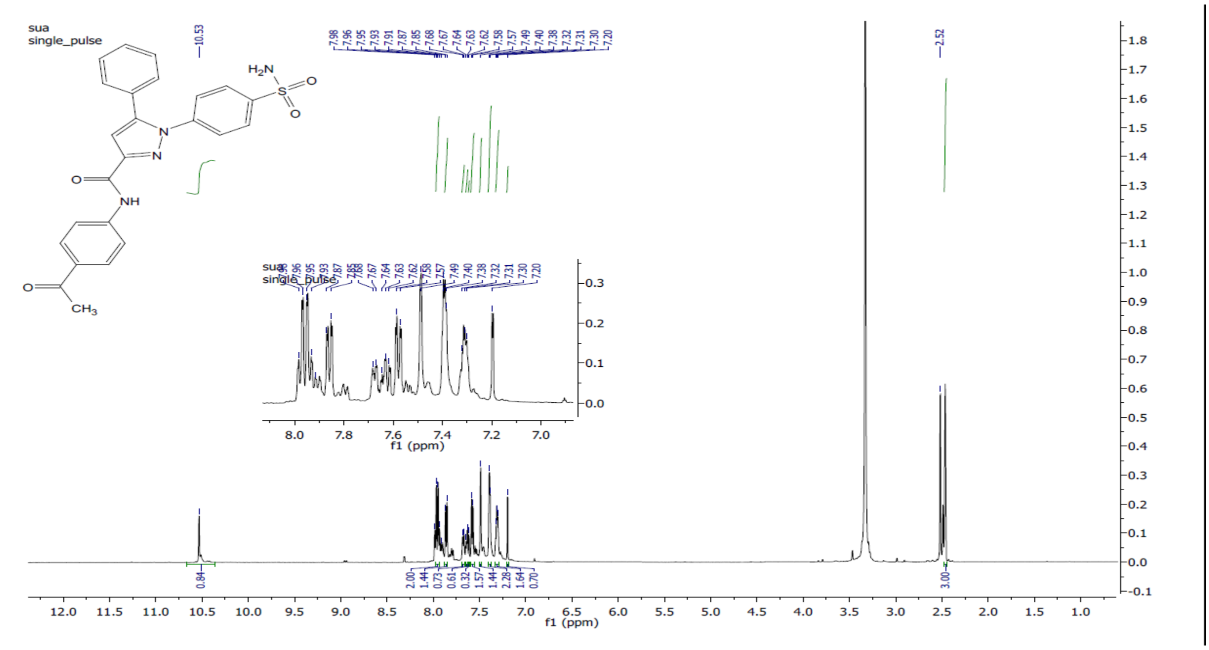

^{1}H-NMR of compound **9a** in DMSO-*d6*.

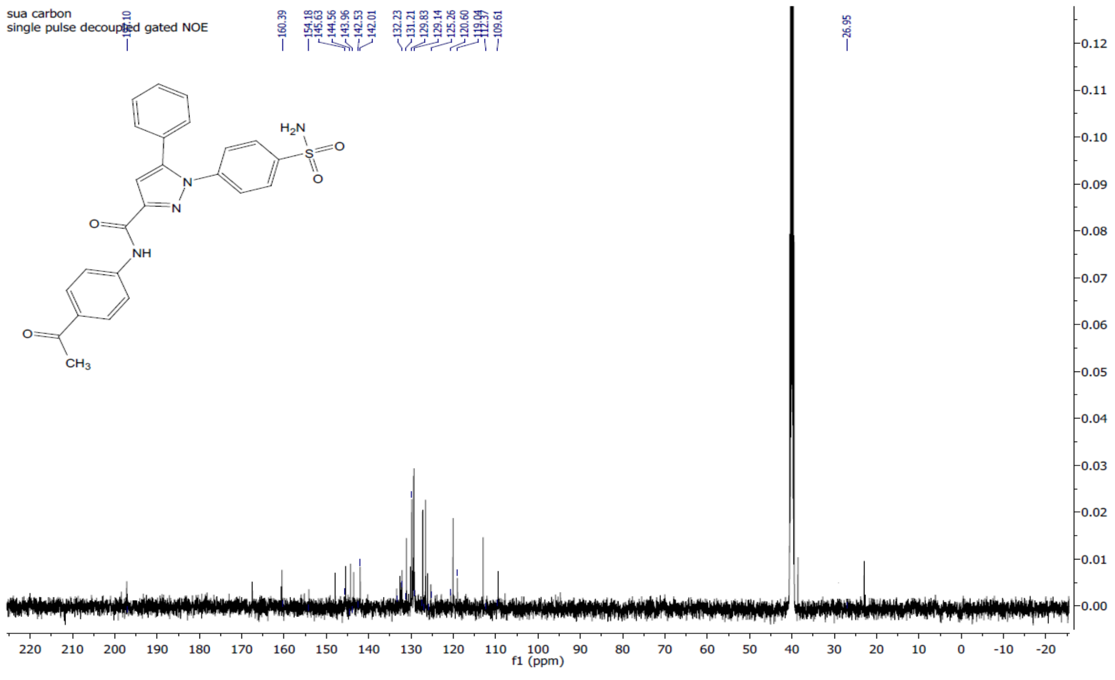

^{13}C-NMR of Compound **9a** in DMSO-*d6*.

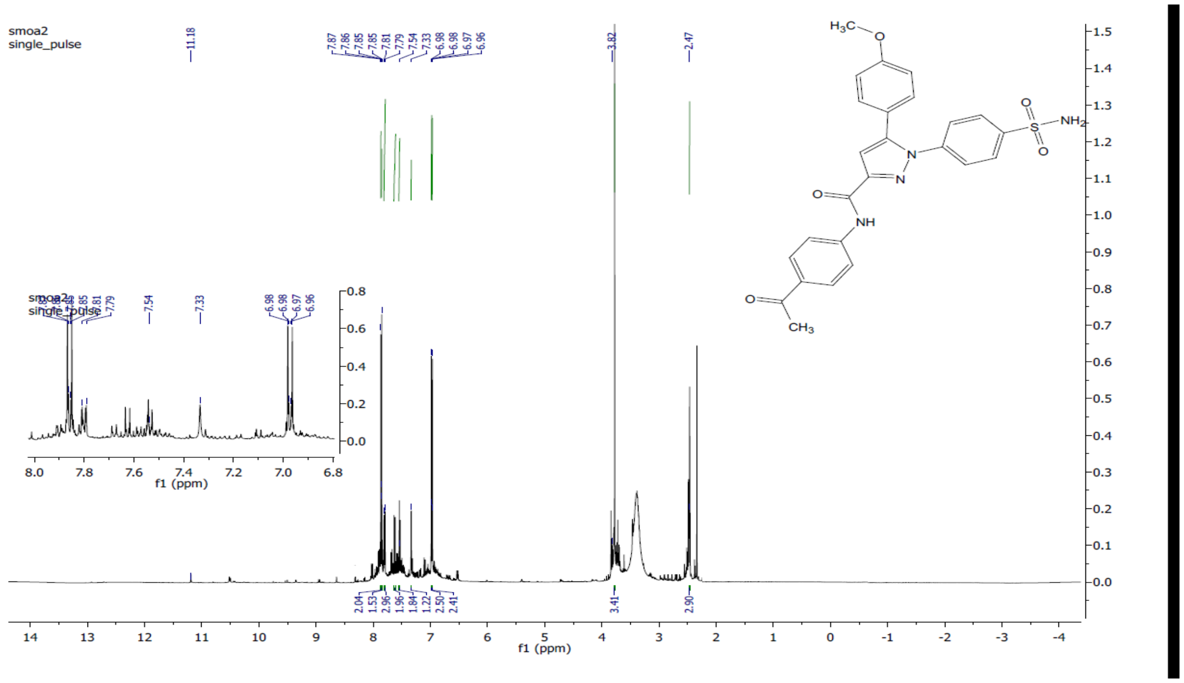

^{1}H-NMR of compound **9b** in DMSO-*d6*.

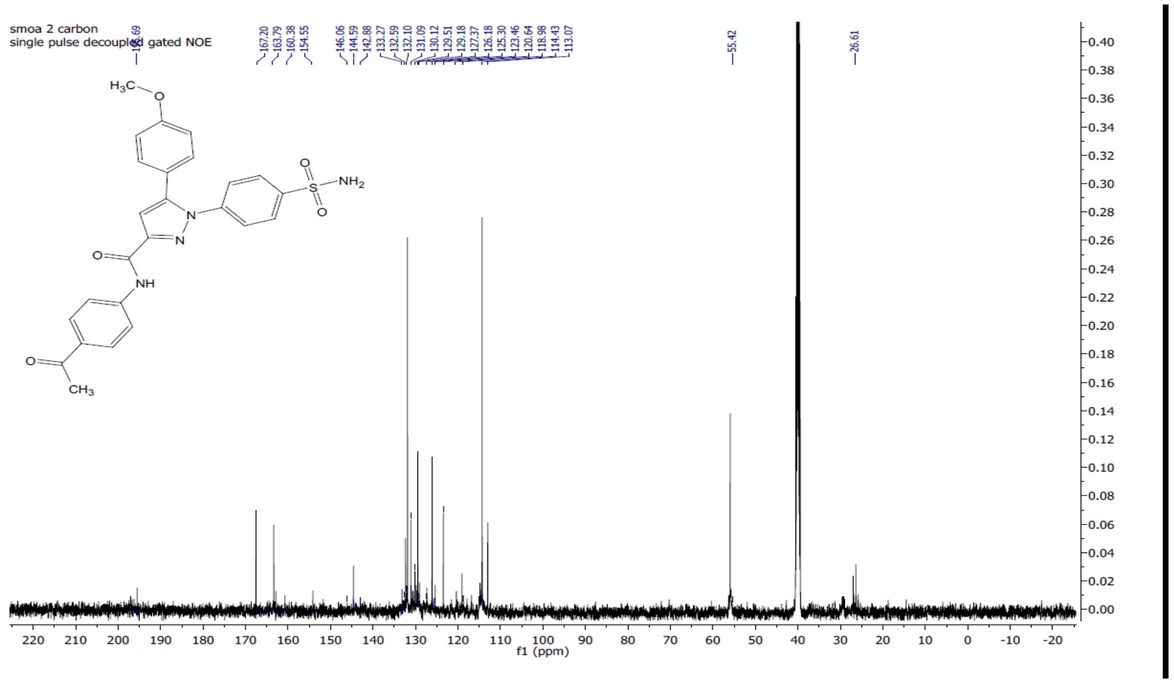

^{13}C-NMR of Compound **9b** in DMSO-*d6*.

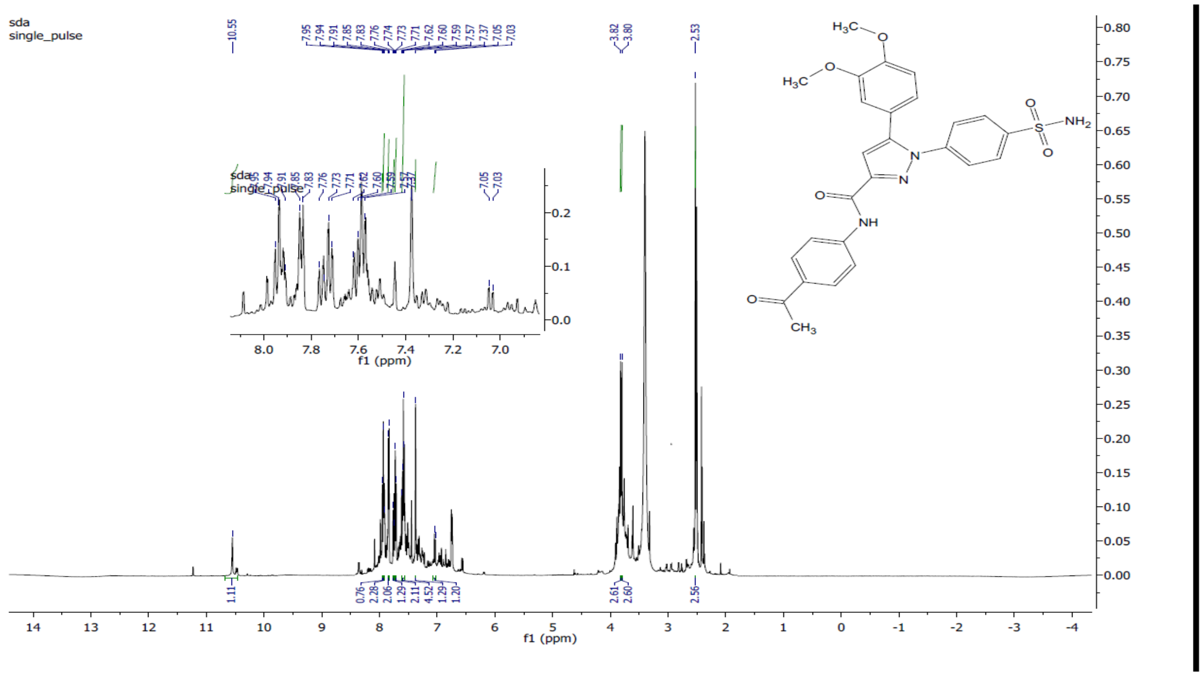

^{1}H-NMR of compound **9c** in DMSO-*d6.*

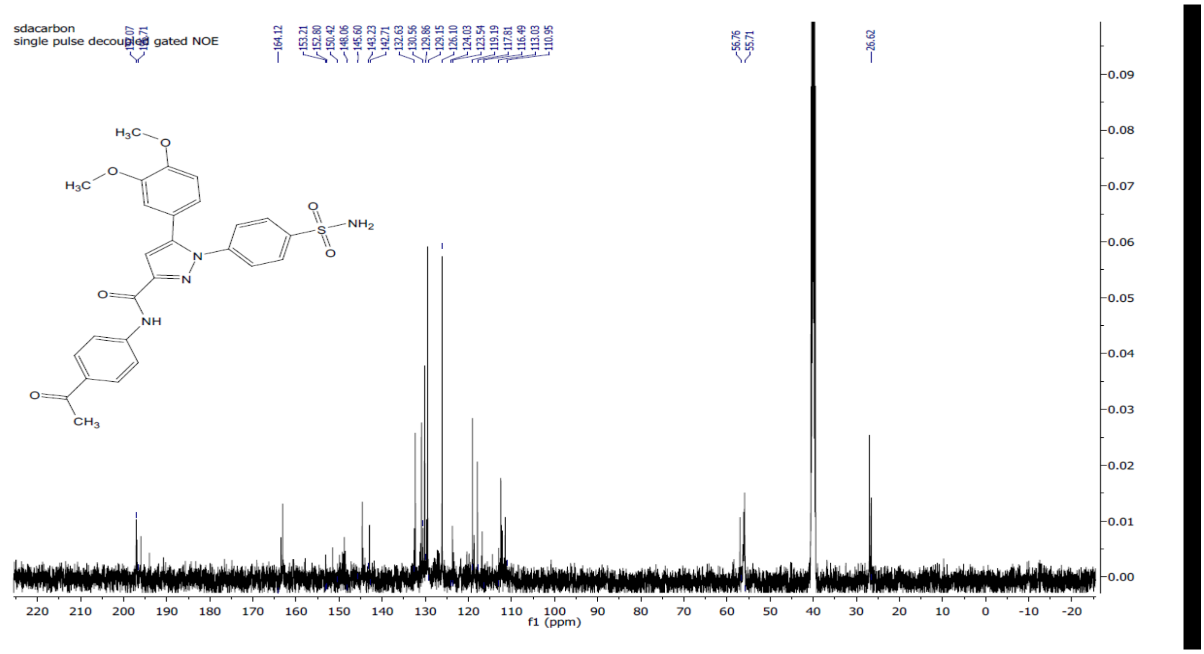

^{13}C-NMR of Compound **9c** in DMSO-*d6*.

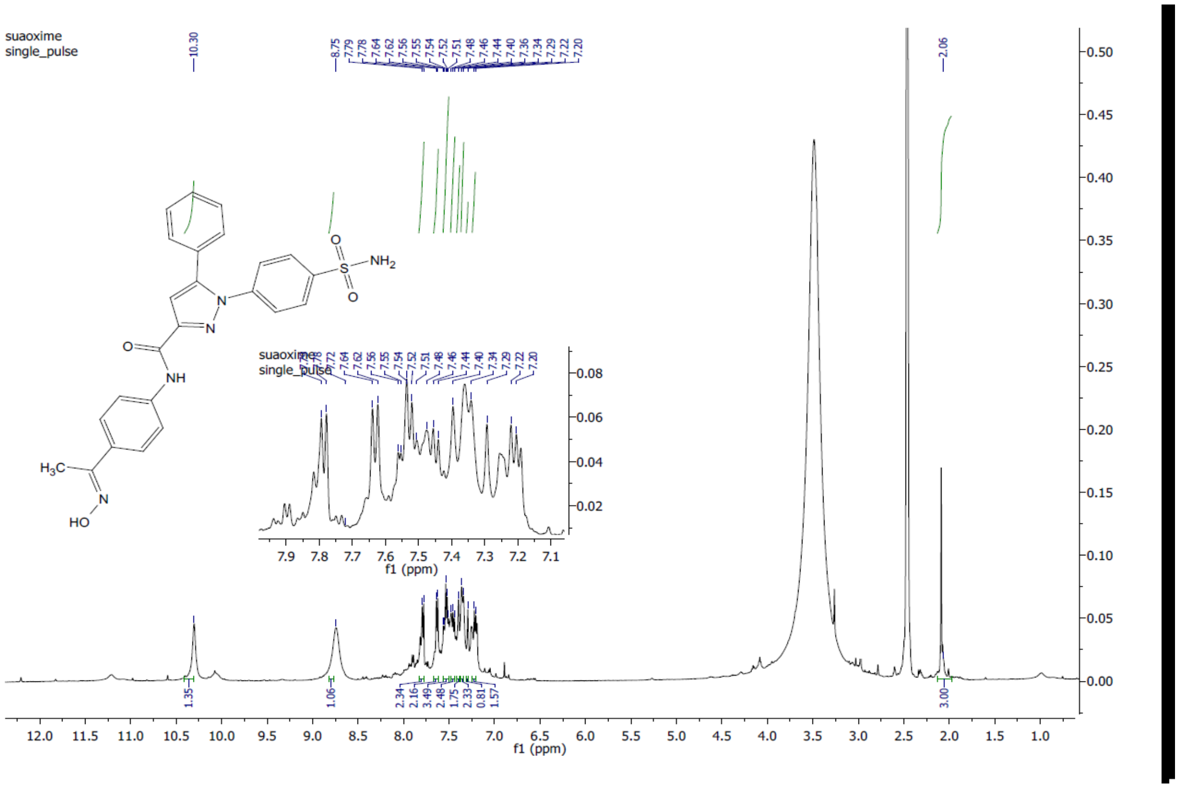

^{1}H-NMR of compound **10a** in DMSO-*d6*.

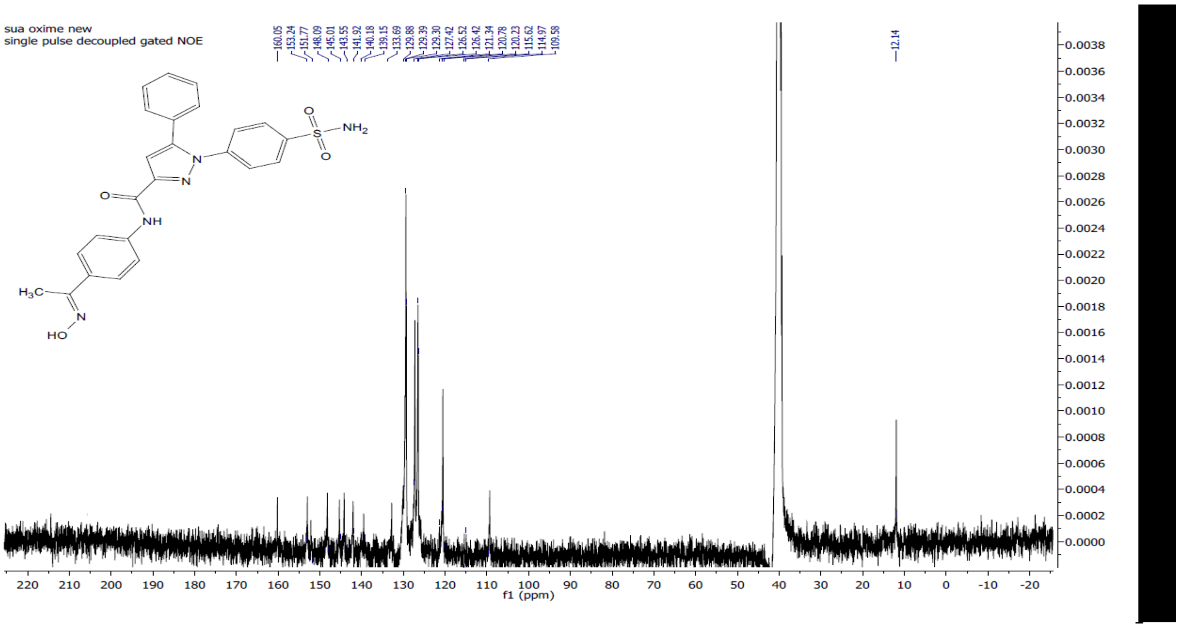

^{13}C-NMR of Compound **10a** in DMSO-*d6*.

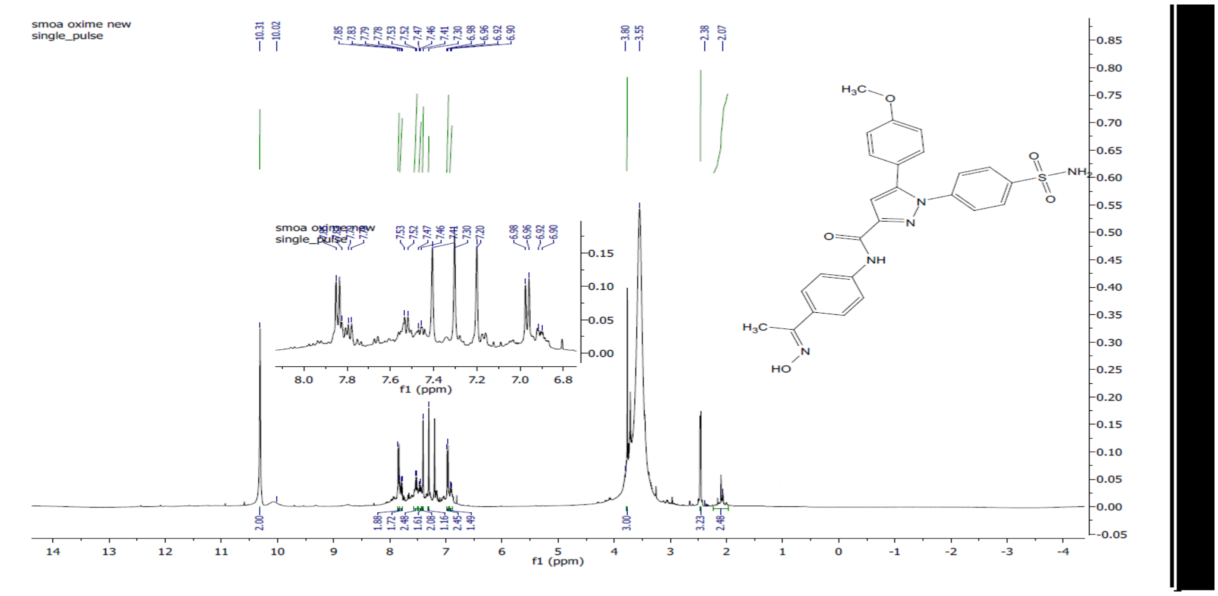

^{1}H-NMR of compound **10b** in DMSO-*d6*.

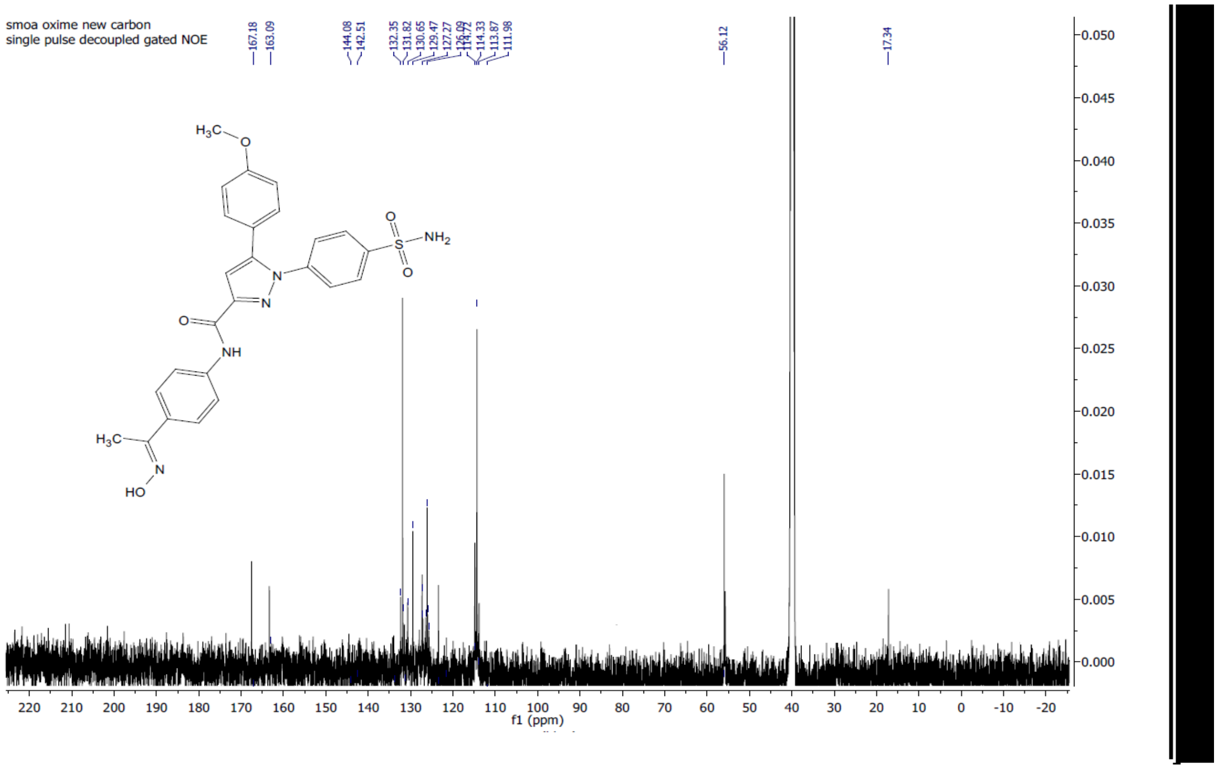

^{13}C-NMR of Compound **10b** in DMSO-*d6*.

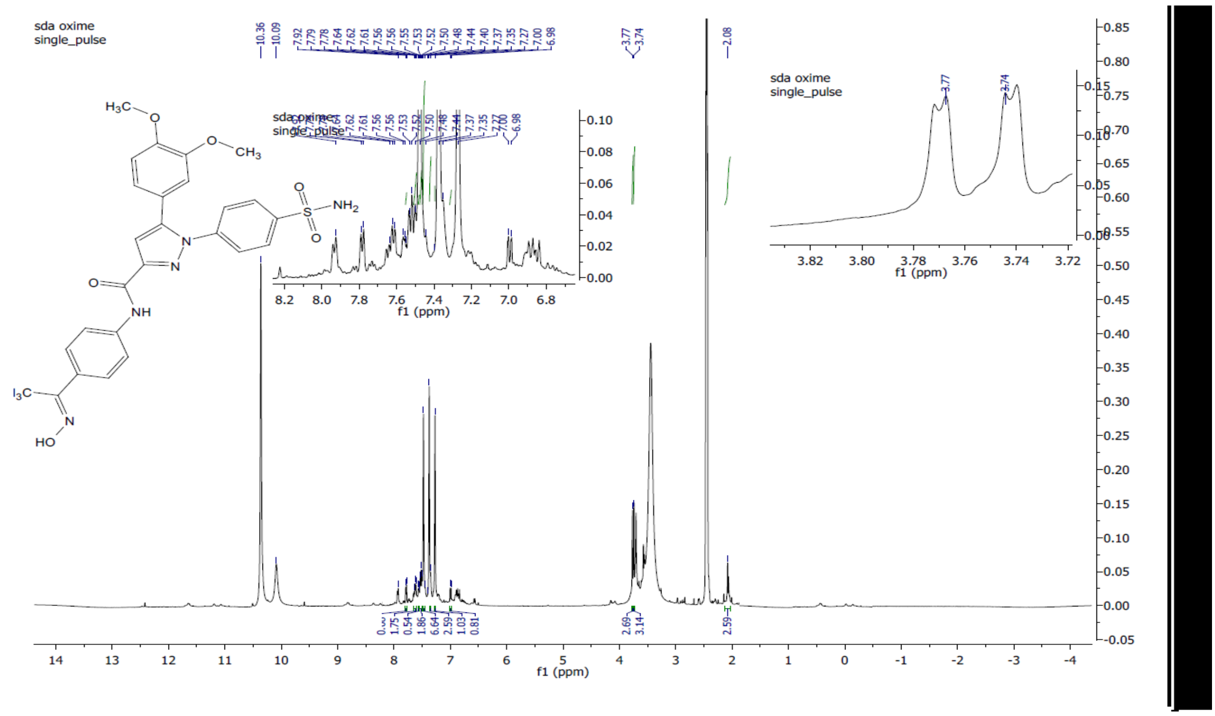

^{1}H-NMR of compound **10c** in DMSO-*d6*

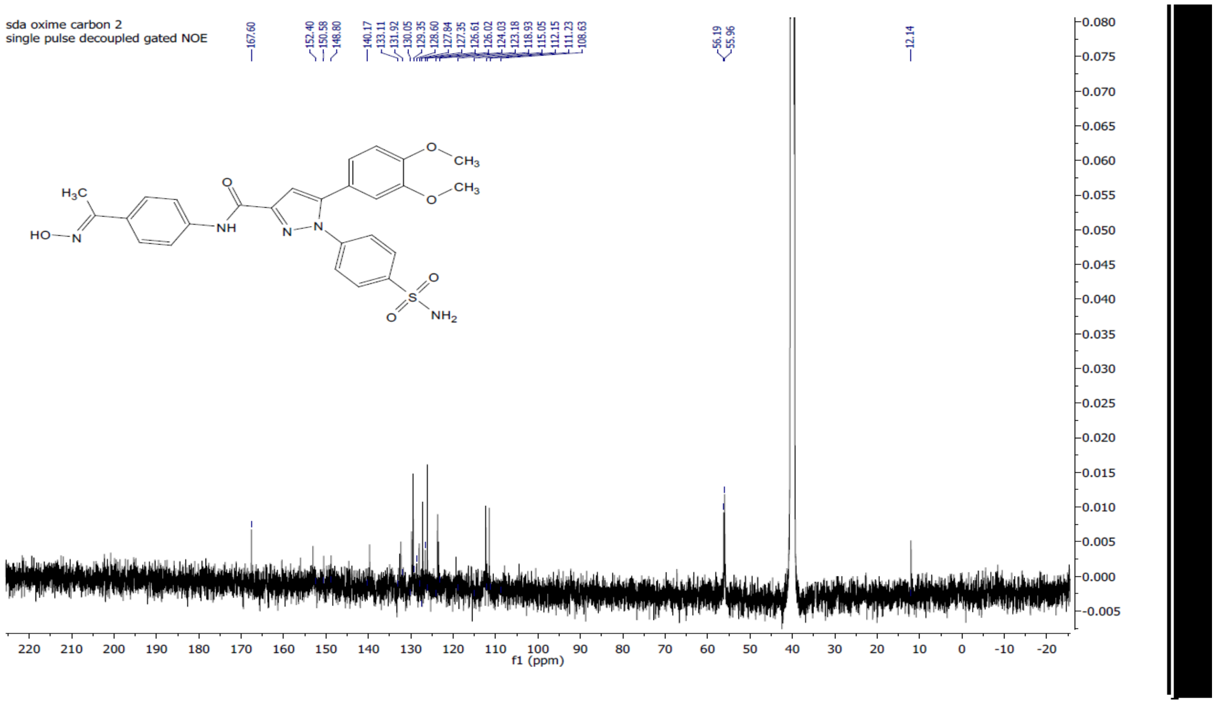

^{13}C-NMR of Compound **10c** in DMSO-*d6*.
